# Addressing Patient Specificity in the Engineering of Tumor Models

**DOI:** 10.3389/fbioe.2019.00217

**Published:** 2019-09-12

**Authors:** Laura J. Bray, Dietmar W. Hutmacher, Nathalie Bock

**Affiliations:** ^1^School of Chemistry, Physics and Mechanical Engineering, Science and Engineering Faculty, Institute of Health and Biomedical Innovation, Queensland University of Technology, Brisbane, QLD, Australia; ^2^Centre in Regenerative Medicine, Institute of Health and Biomedical Innovation (IHBI), Queensland University of Technology (QUT), Kelvin Grove, QLD, Australia; ^3^Translational Research Institute, Queensland University of Technology (QUT), Brisbane, QLD, Australia; ^4^School of Biomedical Sciences, Faculty of Health and Australian Prostate Cancer Research Centre (APCRC-Q), Brisbane, QLD, Australia; ^5^Australian Research Council (ARC) Industrial Transformation Training Centre in Additive Biomanufacturing, Queensland University of Technology (QUT), Kelvin Grove, QLD, Australia

**Keywords:** tumor heterogeneity, tumor microenvironment, 3D tumor models, primary cells, patient-derived, tissue engineering, hydrogels, microfluidics

## Abstract

Cancer treatment is challenged by the heterogeneous nature of cancer, where prognosis depends on tumor type and disease stage, as well as previous treatments. Optimal patient stratification is critical for the development and validation of effective treatments, yet pre-clinical model systems are lacking in the delivery of effective individualized platforms that reflect distinct patient-specific clinical situations. Advances in cancer cell biology, biofabrication, and microengineering technologies have led to the development of more complex *in vitro* three-dimensional (3D) models to act as drug testing platforms and to elucidate novel cancer mechanisms. Mostly, these strategies have enabled researchers to account for the tumor microenvironment context including tumor-stroma interactions, a key factor of heterogeneity that affects both progression and therapeutic resistance. This is aided by state-of-the-art biomaterials and tissue engineering technologies, coupled with reproducible and high-throughput platforms that enable modeling of relevant physical and chemical factors. Yet, the translation of these models and technologies has been impaired by neglecting to incorporate patient-derived cells or tissues, and largely focusing on immortalized cell lines instead, contributing to drug failure rates. While this is a necessary step to establish and validate new models, a paradigm shift is needed to enable the systematic inclusion of patient-derived materials in the design and use of such models. In this review, we first present an overview of the components responsible for heterogeneity in different tumor microenvironments. Next, we introduce the state-of-the-art of current *in vitro* 3D cancer models employing patient-derived materials in traditional scaffold-free approaches, followed by novel bioengineered scaffold-based approaches, and further supported by dynamic systems such as bioreactors, microfluidics, and tumor-on-a-chip devices. We critically discuss the challenges and clinical prospects of models that have succeeded in providing clinical relevance and impact, and present emerging concepts of novel cancer model systems that are addressing patient specificity, the next frontier to be tackled by the field.

## The Heterogeneity of Cancer

**Figure 1:**
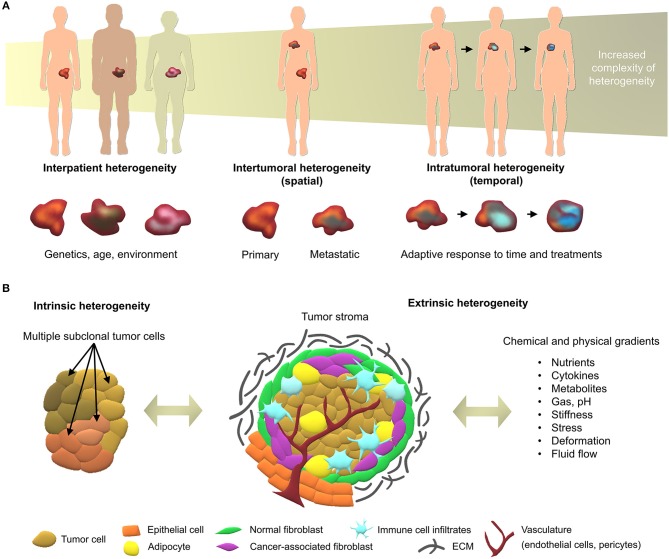
Overview of cancer heterogeneity types. **(A)** Tumors vary according to the characteristics of patients and location in the body, along with time and treatments. **(B)** Local heterogeneity arises from genetic/epigenetic intrinsic factors, stromal extrinsic factors, and chemical/physical factors, which, combined, contribute to the complexity of tumor microenvironments.

The multi-faceted nature of cancer as a dynamic disease makes it complex to fully capture the traits of individual tumors at specific points in time (Dagogo-Jack and Shaw, 2017). With a high number of different cancer types and sub-types, interpatient heterogeneity arises due to unique genetics and epigenetics, as well as dynamic factors such as age, environment, lifestyle, and medical history (Alizadeh et al., 2015). Intertumoral and intratumoral heterogeneity further increase during the course of the disease (Figure 1), upon degree, stage, and treatment history which, ultimately, lead to therapeutic resistance and treatment failures in patients (Fisher et al., 2013). With the continual biotechnological advances that enable in-depth sequencing, specific tumor subclones may be isolated and used in tumor models of heterogeneity, representing the next roadblock to tackle in order to develop more effective personalized medicine (Lawson et al., 2018).

At the tumor level, heterogeneity arises from two key players; the genetic/epigenetic intrinsic factor and the extrinsic stromal factor (Lawson et al., 2018). Intrinsically, variations in clonal growth, functional properties, metabolic state, and expression markers are commonly found within the same tumor clones (Burrell et al., 2013; Sabaawy, 2013). The clonal evolution model is the most accepted cause of intratumoral heterogeneity, where genetic/epigenetic alterations lead to novel clones with better advantages compared to ancestral clones (Burrell et al., 2013). Although debated, cancer stem cells may further increase heterogeneity through epigenetic variations, which give rise to small subpopulations within tumors (Shackleton et al., 2009). Extrinsically, the tumor microenvironment comprises stromal components in various differentiation states, pro/anti-tumor immune products, and the expression of organ-specific extracellular matrix (ECM) (Junttila and de Sauvage, 2013). While tumor cells initially modulate the local tumor microenvironment, activated stromal cells, in turn, generate a feedback loop that contributes to oncogenic phenotypes of the tumor cells, synergistically fueling intrinsic/extrinsic crosstalk (Plava et al., 2019). Anti-neoplastic drug treatment is the most common route to improve overall survival of cancer patients, yet disease heterogeneity often results in unsuitable or ineffective treatments, and may lead to unnecessary toxic side-effects. In the future, advanced sequencing techniques will enable individual molecular characterization, forming the basis of better therapy selection or personalized medicine (Meijer et al., 2017; Senft et al., 2017). Yet, this undertaking requires the validation of biomarkers prior to their implementation in the clinic using patient-specific models that account for both intrinsic and extrinsic heterogeneity factors, in spatial and temporal contexts (Dagogo-Jack and Shaw, 2017).

In this review, we present an overview of the key heterogeneity components of various microenvironments, followed by a discussion of the current patient-specific culture systems that are addressing tumor heterogeneity by using patient-derived cells arising from both tumor and stroma. Finally, we present an outlook for the future, predicting what technology platforms will be able to address patient specificity and accurate disease modeling in order to progress basic research and clinical studies alike.

## Overview of the Key Heterogeneity Components in Tumor Microenvironments

The tumor microenvironment is key to cancer progression and tumors cannot survive without the appropriate support of microenvironment-derived factors (Risbridger et al., 2018). To date, the profiling of clinical specimens has identified gene dysregulations, not only in cancer cells, but also in the adjacent stroma (Planche et al., 2011). Tumor identity is dynamically shaped by physical and chemical parameters arising from cancer/stroma interactions and strongly dictate clonal reprogramming leading to heterogeneous adaptive cellular responses in both tumor and stromal cells (Figure 1B). Hereafter we introduce the key components of heterogeneity, providing background for efficient tumor modeling of patient-specific microenvironments.

### Key Cellular Components

Tumors commonly consist of heterogeneous cell populations that encompass both genetically mutated and unmutated sub-populations (Burrell et al., 2013). Broadly, the cellular stroma contains epithelial cells (Thiery and Chopin, 1999), normal and cancer-associated fibroblasts (CAFs) (Kalluri, 2016), endothelial cells (Hida et al., 2018), adipocyte cells (Cozzo et al., 2017), infiltrating immune cells (Smith and Kang, 2013), and pericytes (Paiva et al., 2018), which assist cancer progression in various ways (Junttila and de Sauvage, 2013). Critical to tumor growth and dissemination, induced angiogenesis is the key component that transcends all cancers (Hanahan and Weinberg, 2011). Metabolic stresses on tumor cells signal for the recruitment of endothelial cells and fibroblasts and the establishment of new microvessels around the tumor stroma, known as the angiogenic switch (Qiao and Tang, 2018). When tumor angiogenesis occurs through this mechanism, the vessels are often irregular, leaky and do not form organized capillaries (Shchors and Evan, 2007). Variability also arises among different organs, where organ-specific endothelial cells influence tumor progression to different extents (Peela et al., 2017).

CAFs are another key stromal component highly responsible for tumor heterogeneity (Ochiai and Neri, 2016). CAFs arise from the secretion of pro-fibrotic cues, such as growth factors, cytokines, and metabolites, following cancer-stroma crosstalk, where myofibroblasts develop from stromal fibroblasts, ultimately leading to a CAF phenotype (LeBleu and Kalluri, 2018). CAFs can also arise from vascular smooth muscle cells, pericytes, circulating fibrocytes and bone marrow derived cells (Ochiai and Neri, 2016). Highly proliferative, CAFs are the largest contributor to ECM remodeling and the main source of collagen production, providing cancer cells with the mechanical support needed for progression (Ochiai and Neri, 2016). The biological properties of CAFs are heterogeneous and different types of CAFs make distinct functional contributions (Junttila and de Sauvage, 2013). CAFs are also key to metastasis success and a fraction can disseminate along with cancer cells, helping to prepare the secondary microenvironment for cancer cell homing and survival and overall contribute to high levels of heterogeneity (LeBleu and Kalluri, 2018).

During progression of the primary tumor, cancer cells may disseminate throughout the body using blood or lymphatic vessels, or may advance via direct invasion of surrounding microenvironments (Stacker et al., 2002). Cell-cell and cell-matrix interactions, and paracrine signaling are key to these activities (Lu et al., 2012). Cancer cell migration itself is controlled through a paracrine loop involving colony stimulating factor 1 (CSF1), epidermal growth factor (EGF), and their receptors, which are differentially expressed on carcinoma cells and macrophages, resulting in movement of cancer cells toward macrophages (Smith and Kang, 2013). Additional paracrine loops exist between cancer cells expressing C-X-C chemokine receptor 4 (CXCR4) and stromal cells, such as fibroblasts and pericytes, producing the stromal cell-derived factor 1, also known as C-X-C motif chemokine 12 (CXCL12), which contribute to directional cancer cell migration (Kucia et al., 2005). Cancer cell intravasation into the blood circulation is directly associated with the presence of perivascular macrophages and tumor associated macrophages (Jeffrey et al., 2004; Wyckoff et al., 2007). The macrophages, along with the cancer cells themselves, mediate disruption in the vascular basement membrane (Bissell and Radisky, 2001). Entry of cancer cells into the lymphatic system is due to a lack or disruption in the basement membrane, as well as help from factors secreted by neighboring pericytes, among other influences (Saharinen et al., 2004). Intrinsic to tumor cells, epithelial-to-mesenchymal transition (EMT), and reverse EMT, are the key cellular processes for tumor progression and survival in the secondary microenvironment, by modulation of E-cadherins (Yao et al., 2011; Banyard and Bielenberg, 2015; Paduch, 2016).

The formation of a pre-metastatic niche is required to facilitate tumor cell engraftment and is formed due to the secretion of factors from the tumor itself (Kaplan et al., 2005; Hiratsuka et al., 2006; Psaila and Lyden, 2009). Pre-metastatic niches are intrinsic to each cancer and are proposed as a key determinant to the site of extravasation (Chen et al., 2018). Attracted by local factors, hematopoietic progenitor cells, stromal cells, endothelial cells, and macrophages aggregate at the pre-metastatic niche (Kaplan et al., 2005; Hiratsuka et al., 2006). After surviving in the circulatory microenvironment, only around 0.01% of extravasated tumor cells home to the pre-metastatic niche (Chambers et al., 2002; Kaplan et al., 2006). While some cells will remain dormant or die shortly after homing, surviving cells start to heavily modify the ECM, forming micrometastases (de Boer et al., 2009), which are too small to be captured by current detection methods. The growth and maintenance of metastatic tumors is due to tumor cell clonal adaptation to the new environment and help from the local cellular populations, ECM produces and dynamic paracrine signaling (Psaila and Lyden, 2009). While there may be some level of genomic concordance between primary tumors and metastatic tumors in some cancers (e.g., colorectal; Urosevic and Gomis, 2018), heterogeneity is, overall, highest in metastatic tumors (Fidler, 1978). This is due to having resided longest in the patient, leading to a high number of subclonal evolutions and exposure to multiple microenvironments, further altering cellular programs to better-fit each site specifically (Dagogo-Jack and Shaw, 2017). Importantly, as cellular heterogeneity increases steadily as a tumor progresses, cellular/non-cellular interactions and their variable physicochemical gradients further contribute to heterogeneity, progression, and therapy response (Burrell et al., 2013).

### Key Non-cellular Components

The ECM is a key player in regulating cancer cell behavior by offering both biophysical and biochemical cues that influence cancer cell proliferation, invasion, migration, differentiation, metastasis, therapy response, and apoptosis (Griffith and Swartz, 2006). The ECM is highly dynamic and heterogeneous, structurally and biochemically, hence heavily contributing to the heterogeneity of cancer microenvironments (Seewaldt, 2014). The ECM comprises several hundreds of macromolecule types (Filipe et al., 2018), such as collagens, proteoglycans, elastin, fibronectin, laminin, hyaluronan, and is remodeled by enzymes such as matrix metalloproteinases (MMPs) (Lu et al., 2012). Inflammation involves high ECM remodeling with large ECM protein deposition, which are crosslinked by increased levels of lysyl oxidase (LOX) (Barker et al., 2012), contributing to solid stresses (Kalli and Stylianopoulos, 2018), tumor ECM stiffening (Gkretsi and Stylianopoulos, 2018), and drug resistance (Erler et al., 2006). The increased deposition of ECM proteins promotes cancer progression by altering cell-cell adhesion, cell polarity and growth factor signaling (Walker et al., 2018). A review by Poltavets et al. describes the role of each cell type in directing ECM change and how this influences cancer cells and their plasticity (Poltavets et al., 2018). The ECM organization is different for each tumor microenvironment, including large variations in stiffness, topography, and biochemical composition (Filipe et al., 2018). Highly aligned fiber networks are found in connective tissues such as bone, while amorphous substrates are found in disorganized structures, as seen in the brain, resulting in higher and less stiff microenvironments, respectively (Malandrino et al., 2018). As an example, brain is in the 100–2,000 Pa range (Cox and Erler, 2011; Barney et al., 2015) and glioblastoma-associated ECM is mostly composed of collagen IV, procollagen III, laminins, fibronectin, and hyaluronan (HA)-fibrillar collagens (Gkretsi et al., 2015). Conversely, the normal glandular tissue of breast is in the 1–45 kPa range (Cox and Erler, 2011; Ramião et al., 2016) and tumor ECM involves collagen I, IV, V, fibronectin, laminins, entactin, proteoglycans, and glycosaminoglycans (Gkretsi et al., 2015). Tumor ECM has a unique protein composition which, when isolated, has been shown to enhance the growth of cancer cells *in vitro*, compared to normal ECM (Romero-López et al., 2017). Stiffness increases dramatically during cancer, for example a 13-fold increase in stiffness was observed from fibroglandular breast tissue to high-grade invasive ductal carcinoma (Samani et al., 2007). In turn, increased stiffness reciprocally forces tumor progression (Boyd et al., 2014). Increasing ECM stiffness in breast cancer tissues in particular is a prominent indicator for cancer aggressiveness, metastatic potential, response to therapy, and overall prognosis (Acerbi et al., 2015). This is linked to ECM changes in both tissue organization and composition with matrix proteins such as increasingly crosslinked fibrillar collagens, fibronectin, laminins, proteoglycans, as well as remodeling enzymes (Insua-Rodríguez and Oskarsson, 2016).

Cancer invasion is critically prompted by the tumor ECM, which deposition is increased compared to normal stroma, resulting in higher matrix stiffness and cancer cell migration by durotaxis (Friedl and Alexander, 2011). A disruption in intercellular adhesion results in the detachment of certain tumor cells from the primary mass. These cells then migrate through the ECM, invading surrounding tissue and leading to the degradation of natural ECM. Collagen fibers are often used by cancer cells for this purpose, via microtrack formation (Paul et al., 2017). As these fibers are often attached to the local blood vessels, cancer cells can collect at these sites (Condeelis and Segall, 2003). Initially, the collagen fibers found in primary tumors progressively align themselves perpendicularly to the tumor boundaries, facilitating dissemination from the primary site (Belgodere et al., 2018). When cancer cells eventually detach from the primary tumor and become motile, they undertake migration by heterogeneous modes, namely mesenchymal or amoeboid (Malandrino et al., 2018). Recent studies suggest that while migration starts as a collective of tumor cells, eventually cell migration becomes an individual process facilitated more by the actin cytoskeleton and less by their arrangement along the collagen fibers (Ilina et al., 2018). In other cases, even though a degree of porosity at the micrometer scale exists within anatomical structures, cancer cells need to degrade surrounding ECM when the pore size is <7 μm^2^ (Wolf et al., 2013). The tumor cells may then intravasate into blood or lymphatic vessels entering the circulation, which can happen both actively or passively (Diab et al., 2009). Intravasation is usually favored chemically by chemokine gradients that actively lead cancer cells toward circulatory vessels, yet it can also take place due to high local stresses and a fragile vascular network that ultimately passively collapses (Peela et al., 2017). There is a definite role for protein assembly from the stromal compartment in influencing tumor cell colonization, including fibronectin, collagen IV, tenascin, and periostin, which are deposited by fibroblasts and endothelial cells (Barkan et al., 2010; Oskarsson, 2013). These proteins promote cell adhesion and growth at the metastatic sites. It has also been hypothesized that integrin expression is an important factor in the targeting of an organ by a tumor cell. Integrin β1, α2, and α6 are expressed in the brain, liver, and lung ECM, and overall have control over cell adhesion in these sites (Barney et al., 2015). Moreover, a role for exosomes, also known as extracellular vesicles which carry signaling molecules, has been defined in the formation of the pre-metastatic niche by preparing the tissue for extravasated tumor cell propagation (Costa-Silva et al., 2015; Hoshino et al., 2015). The exosomes derived from tumor cells show integrin expression that promotes binding to organ-specific cells (Hoshino et al., 2015). Once the tumor cells arrive, they are then maintained in a fibronectin and growth factor rich pre-metastatic niche. The remodeling of local tissue after tumor cell arrival is essential to manage invasion and metastatic outgrowth (Paget, 1989). Therefore, expression of MMPs are also upregulated in the pre-metastatic niche (Kaplan et al., 2005). ECM composition and mechanical stiffness are equally remodeled heavily around metastatic tumors. Metastases usually have more aggressive features compared to primary tumors, with more active paracrine signaling for more rapid growth at the secondary site (Urosevic and Gomis, 2018). Various cancer types and subtypes preferentially metastasize to different organs, suggesting that each cancer is more inclined to home to and grow in a distinct microenvironment (Minn et al., 2005; Bos et al., 2009; Peinado et al., 2017). As a result, the identification of major ECM components for each tumor microenvironment, their biochemical composition, spatial organization, and resulting stiffness provide a relevant foundation to engineer more physiologically-relevant matrices, in turn better addressing tumor ECM heterogeneity.

## Engineering Patient-Specific Tumor Microenvironment Models

**Figure 2:**
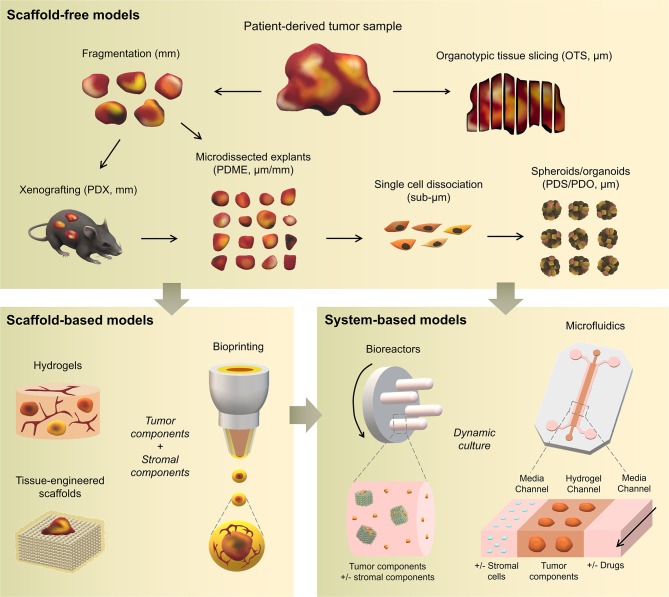
Overview of patient-specific tumor models. Traditionally used with no matrix or simple natural matrices, and mainly for drug testing purposes, patient-derived materials are now used in combination with scaffold-based biomaterials, allowing the incorporation of stromal components to better mimic the native microenvironment or to study a specific process (angiogenesis, metastasis). Both approaches are also being used with dynamic systems to; further mimic/test physical and chemical gradients, better control the addition of stromal components, increase viability, and enable multiple drug testing.

Traditional three-dimensional (3D) tumor culture systems have relied on immortalized cell lines. While cell lines are essential to validate the efficiency of novel culture systems and provide important insight in tumor behavior when grown in 3D, they lack power as tumor models for personalized medicine. For instance, even if cancer cell lines retain driver mutations, several studies revealed a drift at the transcriptomic level where cancer cell lines bore more resemblance to each other, regardless of the tissue origin, than to the clinical samples they were modeling (Gillet et al., 2013). Hence, the use of cell lines, even in *in vivo* preclinical 3D settings, has failed to be an efficient therapy platform for patients. This has correlated with high drug failure rates in phase II and III clinical trials (Colditz and Peterson, 2018), calling out for a paradigm shift toward the use of patient-derived cells. Yet, the culture of such cells *in vitro* is challenging due to difficulties in isolation, low isolated numbers, and limited proliferative capacity due to being highly dependent on the supportive surrounding stroma. Where successful two-dimensional (2D) culture of these cells allows rapid diagnostic testing at low passages, extended culture is impossible, and whereas they are more relevant than cancer cell lines, they are not suited to the wide testing span required to be an effective predictive model. Yet drug efficacy prediction is not always the goal and an important consideration lies in a model's purpose, where model complexity is largely dependent on the objectives (Katt et al., 2016). While some of the simpler systems are most suited for drug screening, the more complex and physiologically relevant models are necessary for validation purposes (Meijer et al., 2017). Primary culture systems in 2D have so far remained optimal for drug screening, as they provide high-throughput possibility. However, the local penetration of drugs in a real tumor is influenced by interstitial fluid flow, hypoxia, pH, and ECM composition (Vilanova et al., 2018) that are missing in the 2D setting, leading to less therapeutic efficacy correlation and a reduced ability to serve as drug efficacy predictors *in vivo*. The development of more advanced 3D systems is tackling some of these issues, yet to date, there are no pre-clinical models that fully recapitulate the patient-specific stromal, immune, structural, chemical, and molecular aspects of the heterogeneous microenvironments that cancer cells are sequentially exposed to in the course of the disease (Belgodere et al., 2018). This concept also needs to be balanced with over-engineering considerations, where a complex model may not be as easily translated for routine pre-clinical use, but may serve as a relevant mechanistic platform. Nevertheless, current advances have started to recapitulate more complex stages of cancer progression, integrating advanced biomaterials, and technologies, which current state-of-the-art will be discussed hereafter. We will describe how patient-derived microenvironments are more traditionally modeled by scaffold-free approaches, followed by novel biomaterials and tissue engineering techniques that have allowed more complexity. Finally we will discuss the system-based technologies that employ dynamic culture approaches (Figure 2).

### Scaffold-Free Approaches

To date, a large proportion of patient-derived cultures have been used for drug testing purposes, rather than for the recapitulation and study of cancer processes, which are predominantly performed using cell lines. Other than very limited material availability, one of the critical hurdles when dealing with patient-derived materials, resides indeed in maintaining the tissue for a period sufficient to enable drug testing and biological assessment. As such, simple and short-term strategies have been used traditionally and are described hereafter.

#### Patient-Derived Xenografts (PDXs)

PDXs have been standard practice for target validation, proposing, to date, the most advanced preclinical models that can overcome issues from *in vitro* settings. PDXs involve the propagation of a fresh patient tumor biopsy in immunocompromised mice (NOD/SCID, Nude, NSG) in either ectopic or orthotopic sites, including intact stroma and ECM architecture. In some cases, dissociated tumor cells are regrown in organoids using Matrigel® (Kondo et al., 2018) or other gels [fibrin (Liu et al., 2012), gelatin (Kondo et al., 2011)] prior to implantation. The presence of the mouse circulatory system allows the testing of chemotherapeutics, while also monitoring the downstream effects on various organs. The tumors of many cancers have been used for PDXs and while some metastatic tumors are increasingly used for PDXs [pancreatic ductal adenocarcinoma (Roife et al., 2016), uveal melanoma (Nemati et al., 2010), colorectal cancer (Bertotti et al., 2011; Julien et al., 2012), breast cancer (Whittle et al., 2015), prostate cancer (McCulloch et al., 2005; Nguyen et al., 2017; Beshiri et al., 2018; Risbridger et al., 2018)], a large focus has been on primary tumors. Some of the latest studies include xenografting of primary breast cancer (Matossian et al., 2019), glioblastoma (Hribar et al., 2019), head and neck cancer (Majumder et al., 2015; Ghosh et al., 2019), prostate cancer (Fong et al., 2014), pancreatic ductal adenocarcinoma (Roife et al., 2016), and colorectal cancer (Kondo et al., 2011). So far, they have been used for biomarker screening and testing, pre-clinical drug evaluation, and personalized medicine strategies (Hidalgo et al., 2014).

Within the native stroma and architecture, PDXs retain the global biological and genetic characteristics of the native tumor and remain relatively stable over multiple passages. Yet, PDXs present limitations with engraftment rates in mice and cross-species contamination which alter ECM composition, ultimately an important factor altering tumor cells in this long-term incubation setting. Some excised tumors also present with a lack of viable human stroma, which may be rapidly overcome by mouse stroma and can be influenced by the xenograft sites. This is critical for tumor tissues that have low proliferation rates, enabling further colonization by host cells (Risbridger et al., 2018). Depending on the site of implantation and type of tumor (primary, metastatic), some PDXs can be established relatively rapidly [1–3 weeks for glioblastoma PDX (Tentler et al., 2012)] whereas some PDXs require months of culture [up to 22 months for prostate cancer (Risbridger et al., 2018)]. Those significant culture times are problematic as it increases genetic alterations, in turn lengthening drug screening times, altering responses and reducing predictive power. For example, Daniel et al. showed that PDX models of small cell lung cancer (SCLC) retained a gene expression signature similar to primary tumor tissue, yet irreversible changes occurred when brought back in culture and re-established as secondary xenografts (Daniel et al., 2009). As PDXs do not fully account for non-cell autonomous heterogeneity of the tumor microenvironment (Cassidy et al., 2015), various strategies have been used. Specific to the stroma, CAFs, and mesenchymal stem cells (MSCs) confer bulk tumor heterogeneity and these could be implanted alongside the PDX (Augsten, 2014). Using matched patient stromal components provide a more relevant humanized microenvironment, yet it may not be possible to isolate and expand cells quickly enough to ensure viability and engraftment success of the original tumor. Immune infiltration is another important aspect, yet for PDXs, immunodeficient mice need to be used, with strains such as NSG, lacking functional lymphocytes, and macrophages (Choi et al., 2018). This has been addressed by implantation of human CD34+ hematopoietic stem cells which can differentiate into T and B cells. The final consideration is ECM which is tissue-specific, while in PDX models, the commonly used method to increase engraftment efficiency is the murine-basement membrane Matrigel, due to its inherent rich composition of growth factors. Moreover, the models used are often ectopic, and hence comprise altered ECM components. These limitations could be addressed by synthetic hydrogel alternatives with ECM components similar to the target microenvironment, and by using orthotopic sites where possible.

Another way to limit material-induced heterogeneity is to limit the time of PDX culture (which are typically in the range of several months). This was recently addressed (2018) by introducing a new PDX variant referred to as “mini-PDXs,” as a rapid drug sensitivity assay so that patients receive personalized chemotherapy in a clinically relevant time frame. In this model, the tumors were dissociated into single cells and inserted in hollow fiber capsules (OncoVee®, Biotech) before implantation in nu/nu mice and cultured for 7 days under various drug treatments, prior to extraction, tumor cell viability, and tumor cell growth inhibition measurements. Little details about the biocompatible capsules were mentioned in these studies, other than that the pore size allowed the passage of molecules <500 kDa. The mini-PDXs were used with patient-derived tumor cells from gastric, lung, pancreatic cancer tissues (Zhang et al., 2018a), metastatic duodenal carcinoma (Zhao et al., 2018), and gallbladder carcinoma (Zhan et al., 2018). Significant differences in drug responsiveness were observed, yet overall survival was longer in patients in the PDX-guided chemotherapy compared to the conventional chemotherapy group of 12 patients with gallbladder carcinoma patients (18.6 vs. 13.9 months) and so was disease free survival (17.6 vs. 12 months) (Zhan et al., 2018). While encouraging, it is important to note that the cell dissociation and short timeframe prohibited any native stromal structure and no proper 3D structure recapitulation (Zhang et al., 2018a) as seen in traditional PDXs. There are also some ethical concerns about using animals for such short-term experiments, when an *in vitro* explant model could lead to the same results. In the future, a comparative study of the mini-PDXs should be done either with explants or organoids, to prove that the method is more predictive.

Ultimately, PDXs are the most widely accepted pre-clinical platforms that address both the heterogeneity and complexity of the original tumor. However it has been shown that PDXs may also eventually falsely recapitulate original tumor traits, since engraftment and propagation can lead to selective maintenance of cancer cells with the most aggressive phenotypes (Hidalgo et al., 2014). Coupled with the lack of an immune system, a high cost for maintenance and ethical considerations, PDXs may not be the most sensible system to use for drug testing.

#### Patient-Derived Organoids (PDOs) and Spheroids (PDS)

**Figure 3:**
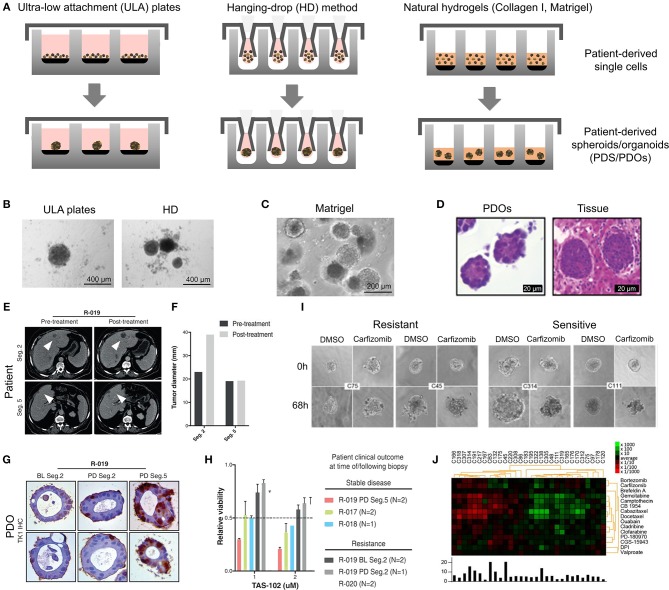
Patient-derived organoids/spheroids (PDO/PDS). **(A)** Schematic diagram displaying the key techniques utilized in current literature for the culture of PDO and PDS. Left, ultra-low attachment (ULA) plates; Center, hanging drop method; Right, natural hydrogels. **(B)** Primary head and neck cancer cells can form consistent spheroids in ULA plates (left), but are not as reproducible in hanging drop culture (right) as visualized using phase contrast microscopy. **(C)** Phase contrast image of a colorectal cancer PDO cultured in Matrigel, and **(D)** hematoxylin and eosin staining comparing PDOs to their matching patient biopsy sample. **(E–H)** PDOs recapitulate intra- and interpatient heterogeneity in response to chemotherapeutics (TAS-102). **(E)** Spheroids were established from a patient with mixed response to TAS-102 with multiple metastases. **(F)** While the segment 2 metastasis rapidly progressed, the segment 5 metastasis remained stable upon TAS-102 treatment. **(G)** Thymidine kinase 1 (TK1) IHC expression is stronger in TAS-102-, compared to sensitive (segment 5) PDOs TAS-102-refractory (segment 2). BL, Core biopsy (baseline); PD, post-treatment (progressive disease). **(H)** There was no significant decrease in cell viability in PDOs in response to TAS-102 in resistant patients. **(I,J)** Diversity of sensitivities for drugs among colorectal cancer tissue-PDX spheroids assessed via high throughput screening. **(I)** Morphological changes after treatment with 100 nmol/L of carfilzomib. **(J)** Heat map and clustering analysis of the average IC50 of 15 drugs in the panel. **(B,C–J)** reproduced with permission from Hagemann et al. (2017), Vlachogiannis et al. (2018), and Kondo et al. (2018), respectively.

PDOs and PDS can arise from dissociated single cells that arrange themselves into a self-directed organizational structure *in vitro* that better retain the characteristics of an original patient tumor compared to 2D monocultures or PDXs (Yuhas et al., 1977; Fischbach et al., 2007). Here, we define PDS as matrix-free 3D cell aggregates and PDOs as 3D cultures supported by naturally-derived matrices. By far the most utilized method of culturing PDOs is using naturally-derived hydrogel matrices, such as Matrigel (Sato et al., 2011; Cheung et al., 2013; Gao et al., 2014; van de Wetering et al., 2015; Weeber et al., 2015; Beshiri et al., 2018; Orditura et al., 2018; Tanaka et al., 2018; Vlachogiannis et al., 2018; Kijima et al., 2019; Mousavi et al., 2019; Schnalzger et al., 2019) or Collagen I (Cheung et al., 2013; Neal et al., 2018), while PDS are often formed using non-adhesive/agarose-coated plates (Bansal et al., 2014; Halfter et al., 2015; Hagemann et al., 2017; Linxweiler et al., 2018) (Table 1), all requiring minimal engineering strategies (Figures 3A–D). PDO cultures using natural hydrogels have long-term culture potential and are unique due to the heterogeneous nature of the tissue from which it is derived. However, challenges arise in the success rate of organoid formation, as PDOs often lack key cellular components that direct intratumoral heterogeneity, such as fibroblasts, immune cells, and other various supporting cell types that contribute to the tumor microenvironment. Nonetheless, compared to cell-line-derived organoids, PDOs have been demonstrated to more accurately maintain the genetic diversity of *in vivo* tumors, more closely recapitulate native histopathology; and can predict *in vivo* drug sensitivity, in turn providing robust pre-clinical models (Nagle et al., 2018).

**Table 1:** Overview of spheroid models.

**Main cancer type**	**Purpose and application**	**Patient numbers**	**Method**	**Maximum culture time**	**References**
Brain cancer	Drug response; preparation of spheroid tissue mircroarray	Not stated	Non-coated well	49 days	Plummer et al., 2019
Breast cancer	Biological studies into the indentification of invasive cancer cells	10	Matrigel and Collagen I Hydrogels	4 days	Cheung et al., 2013
Breast cancer	Drug response in parallel with the clinic	78	Agar-coated plate	5 days	Halfter et al., 2015
Breast cancer	Drug response; biological studies into tumor mutations	27	Matrigel	30 days	Orditura et al., 2018
Esophageal and Oropharyngeal cancer	Biological studies	21	Matrigel	21 days	Kijima et al., 2019
Gastrointestinal cancer	High-throughput drug screening	32	Matrigel	7 days	Kondo et al., 2018
Gastrointestinal cancer	Drug response; mass spectrometry	4	Basement membrane extract	10 days	Liu et al., 2018
Gastrointestinal cancer	Biological studies into tumor mutations	11	Matrigel	10 days	Matano et al., 2015
Gastrointestinal cancer	Biological studies into tumor mutations	26	Matrigel	11 days	Mousavi et al., 2019
Gastrointestinal cancer	Biological studies	20	Matrigel	90 days	Sato et al., 2011
Gastrointestinal cancer	Biological studies	Not stated	Matrigel	As per Sato et al. (2011)	Schnalzger et al., 2019
Gastrointestinal cancer	Biobank establishment; high-throughput drug screening; biological studies into tumor mutations	20	Basement membrane extract	6 days	van de Wetering et al., 2015
Gastrointestinal cancer	Drug response in parallel with the clinic; biological studies	71	Agarose-coated plate and Matrigel	12 days	Vlachogiannis et al., 2018
Gastrointestinal cancer	Biological studies into tumor mutations	14	Matrigel	90 days	Weeber et al., 2015
Head and neck cancer	Drug and radiotherapy response	Not stated	ULA and Hanging-Drop	7 days	Hagemann et al., 2017
Head and neck cancer	Drug response; biological studies	10	Matrigel	30 days	Tanaka et al., 2018
Pancreatic cancer	Biological studies	10	Matrigel	6 months	Boj et al., 2015
Prostate cancer	Biological studies	24	Agarose-coated plate	14 days	Bansal et al., 2016
Prostate cancer	Biological studies	24	Matrigel	3–6 months	Bartucci et al., 2015, 2016
Prostate cancer	Biobank establishment; biological studies	3	Matrigel	14 days	Beshiri et al., 2018
Prostate cancer	Biological studies; establishment of new organoid lines	7	Matrigel	60 days	Gao et al., 2014
Prostate cancer	Drug response	109	ULA plates	Several months	Linxweiler et al., 2018
Prostate cancer	High-throughput drug screening; biological studies	34	Matrigel	12 months	Puca et al., 2018

##### Matrix-free PDS formation

PDS formation without matrix support is most often performed using the Hanging Drop method (Hagemann et al., 2017) and ultra-low-adherent plates/coatings (Bansal et al., 2014, 2016; Halfter et al., 2015; Hagemann et al., 2017; Linxweiler et al., 2018) (Figures 3A,B), and more rarely Aggrewell plates (Hribar et al., 2019). The hanging drop method relies on the gravity-mediated self-assembly of tumor cells, using suspension culture, while ultra-low attachment (ULA) culture plates have surfaces that are not conducive to cell attachment, therefore leading to cellular aggregation. The Aggrewell plates are especially beneficial to obtain highly uniform 3D spheroid cultures. Hagemann et al. (2017) compared the two techniques and found that ULA plates led to more consistent spheroid formation from head and neck squamous cell carcinomas (HNSCCs) than the hanging drop method. A similar protocol using ULA plates was developed for prostate cancer PDS growth using 109 patient samples (Linxweiler et al., 2018). Higher grade Gleason scores led to less spheroid formation than lower Gleason scored tumor tissues. Moreover, tumors of >100 μm often displayed necrosis in their center, mimicking hypoxia, and nutrient deprivation in the early stages of tumor development. PDS were also found to contain and support both prostate epithelial and stromal cells. The models were used to test various drug treatments with results dependent upon individual patient samples. Similarly, Plummer et al. (2019) used a co-culture approach to generate PDS from glioblastoma tissue, first differentiating induced pluripotent stem cells (iPSCs) into neural progenitor cells, and then co-culturing with patient-derived glioblastoma cells, plated on top. After 24 h, both cell types were scraped and re-seeded, prior to exposure to chemotherapeutics or fixed/embedded to create tissue microarrays for high-throughput analyses.

Halfter et al. (2015) reported on a larger scale breast cancer study on the biopsies of 78 patients. PDS were formed using non-treated dishes coated with agar, to prevent cell attachment, leading to cell aggregation. Inter-PDS heterogeneity was noted. Interestingly, the PDS formed were less compact if the tissue was derived from high grade tumors when compared with low grade tumor tissue. The model was able to predict the outcome for various treatments received by individual patients in the clinic. Also using agarose-coated plates, Bansal et al. formed spheroids from both prostate cancer tissues (Bansal et al., 2014, 2016). The authors studied the inhibition of a B-cell-specific insertion site, affecting cell survival, clonogenicity, and motility. Interpatient heterogeneity was observed. Overall, while PDS culture is relatively easy and cost-effective to perform, the biomechanical and biochemical cues provided by a surrounding tissue microenvironment not only affects the development of a tumor, but also the infiltration and effects of various chemotherapeutics. These factors are missing in a matrix-free spheroid model.

##### PDO formation supported by natural matrices

Matrigel, or basement membrane extract, is the most studied matrix to date used to culture PDOs, despite presenting with batch-to-batch variability in manufacturing, and complexity in composition, making it difficult to link matrix signals to cell function (Fang and Eglen, 2017). Hereafter are presented recent or key PDO studies which have used Matrigel. A key paper by Sato et al. (2011) reported the culture of intestinal crypts from 20 patients with colon cancer in Matrigel. The human organoids could be cultured for at least 1 month, after which their morphology changed, and proliferation decreased. The length of PDO culture can be extended with passaging (usually every 1–2 weeks), up to 6 months with the addition of essential growth factors and inhibitors. Subsequently, similar protocols were developed for the culture of pancreatic (Boj et al., 2015), colorectal (van de Wetering et al., 2015; Weeber et al., 2015; Schnalzger et al., 2019), prostate (Bansal et al., 2014; Gao et al., 2014), gastrointestinal (Vlachogiannis et al., 2018), breast (Orditura et al., 2018), and HNSCC (Tanaka et al., 2018; Kijima et al., 2019). PDOs have become a regular tool to expand our knowledge of cancer biology (Matano et al., 2015; Drost et al., 2016). For example, Sato's group later published a report using CRISPR-Cas9 genome-editing to create tumor suppressor and oncogene mutations in normal intestinal PDOs (Matano et al., 2015). These engineered organoids highlighted that these mutations alone were not sufficient to induce cancer progression. Additional studies have sought to apply PDO cultures to drug testing and predictive clinical medicine (Pauli et al., 2017; Kondo et al., 2018; Orditura et al., 2018; Vlachogiannis et al., 2018; Hribar et al., 2019; Kijima et al., 2019) or as biobanks of PDOs for future research (van de Wetering et al., 2015; Beshiri et al., 2018).

Various success rates can be achieved with PDO grown in Matrigel. In pancreatic PDOs (80% success rate), while healthy pancreatic organoids stopped proliferating after 6 months in culture, the tumor samples could be propagated “indefinitely” and survived cryopreservation (Boj et al., 2015). Following orthotopic PDO transplantation into mice, normal ductal architecture within the mouse pancreas was observed and the entire process of tumor development was mimicked. The heterogeneity of the tumor changed over time and tumor progression. Whether these changes were instigated by the organoid itself, the murine microenvironment, or by the Matrigel matrix remains to be determined (Boj et al., 2015). One impressive study characterized a biobank collection of 20 matched patient healthy and malignant colorectal organoids (van de Wetering et al., 2015). Overall, success rate and survival upon freeze-thawing were both ≥80%.

While PDOs are predominantly made of primary tumors, metastatic PDOs remain limited. In metastatic colorectal cancer (Weeber et al., 2015), Matrigel-cultured PDOs (71% success rate) from 14 patients retained 90% of the somatic mutations compared to the original tumors. Kijima et al. successfully developed PDOs from oropharyngeal and esophageal squamous cell carcinomas, highly heterogeneous and therapy resistant cancers (71.4% success rate) (Kijima et al., 2019). Over 3 weeks, the PDOs established mimicry of the original tumor through expression of p53, CD44, proliferation, and autophagy. The PDOs allowed the authors to mimic 5-fluorouracil therapy resistance in those patients associated with high CD44 expression and autophagy. Another study had a high establishment rate of >90% for breast cancer, however this rate dropped during 30 days expansion to ~72% (Orditura et al., 2018). The authors found significant correlation between patients with PI3KA mutations and sensitivity to those inhibitory agents, elegantly addressing interpatient heterogeneity. Perhaps one of the most groundbreaking PDO studies displaying predictive clinical potential, is with PDOs form 110 patients with metastatic gastrointestinal cancer (70% success rate) (Vlachogiannis et al., 2018). Histological evaluations of the PDO and original tissue were similar, and in addition, there was a 96% similarity between the mutational spectrum of the original tumor and the PDO model. Spatiotemporal heterogeneity, and tumor evolution/resistance to treatments, was upheld in the model, with 88% positive predictive value in the clinic (Figures 3E–H) (Vlachogiannis et al., 2018).

Interestingly, prostate cancer has quite a low success rate for PDO propagation. In a long-term prostate PDO cultivation study, the authors compared metastatic tumors, PDX tumors, and PDO models derived from the same patient. Seven PDOs could be maintained for up to 2 months for ~70% of soft metastatic tumor biopsies and ~30% of bone biopsies. However, efficiency of establishing “continuously” proliferative organoid cultures (>6 months) was ~18%. The 3D organoid cultures mimicked the histological structures and marker expression present in the primary patient biopsy specimens, maintaining interpatient heterogeneity (Gao et al., 2014). Another study of HNSCC (Tanaka et al., 2018) also found low (30%) success rates using Matrigel, however the successful PDOs showed similar drug responses as displayed *in vivo*.

PDOs provide a valuable resource in the personalized medicine space and have the potential to model various cancer types. Most crucial when using patient-derived tissues, low tissue quantities can still result in large numbers of testable organoids. The renewable resource that they offer as cryopreserved or live biobanks and the high correlations achieved between treatment response in the clinic and in the organoid model offers a highly accessible tool for drug screening. A key biotechnology development for the application of PDOs for pharmaceutical drug testing is the development of automated pipetting tools that can both create the organoid cultures and apply the drug panel (Kondo et al., 2018), screening thousands of drugs across spheroids (Figures 3I,J). While the variations that occur between batches of Matrigel hinder the reproducibility of the organoid cultures (Postovit, 2016), the engineering of various semi-synthetic and synthetic matrices (Bray et al., 2017, 2018; Romero-López et al., 2017; Wang et al., 2019) may be able to build a new platform from the bottom-up rather than starting with a complex microenvironment such as Matrigel. Moreover, the morphological and phenotypic differences in PDO behavior between Matrigel and collagen hydrogels (Cheung et al., 2013) reiterates that the microenvironmental cues are directing cell response, warranting careful consideration of matrices used. The lack of blood supply is a limitation in the growth potential of the PDO, however this could be brought together and integrated through novel multi-PDO chip-based platforms (Maschmeyer et al., 2015), or through the co-culture of organoid microenvironments (Birey et al., 2017).

#### Patient-Derived Explants

While PDOs exploit cells regrown in 3D, another patient-specific approach consists of culturing the tumor tissue collected upon surgery, either as organotypic explants or as tissue slice cultures. Advantageously, the 3D structure of the tumor remains intact with only macroscopic dissociation. Patient-derived microdissected explants (PDMEs) are usually minced into pieces prior to gentle dissociation into tissue fragments, while patient-derived organotypic tissue slices (OTS) are either sliced manually using a scalpel or using a specialized slicing instrument, such as a vibratome. The morphology, cell proliferation, and viability of tissues can be maintained using these techniques, although for a relatively short time (Davies et al., 2015; Koerfer et al., 2016; Naipal et al., 2016).

##### Patient-derived microdissected explants (PDMEs)

PDMEs are widely used for drug testing purposes. The primary tissue isolated from surgical specimens is mechanically disaggregated and mildly processed using enzymatic and collagenase digestion. Density centrifugation or sieving may then allow the isolation of micro- to milli-sizes fragments [40–100 μm fractions (Aref et al., 2018; Jenkins et al., 2018; Wang et al., 2018a), 300 μm (Holton et al., 2017), 1 mm^3^ (Moore et al., 2018), 3 mm^3^ (Carr et al., 2014; Cheah et al., 2017)]. A major benefit is that PDMEs do not require days or weeks of tissue manipulation, which is critical to rapid drug screening capabilities. Contrary to PDOs, which may be equally used for drug testing as well as mechanistic investigation, PDMEs have low proliferation indexes and cannot be cultured for more than several days, hence are usually not used for mechanistic investigation. Yet, because of ease of manipulation, their viability can be improved by systems such as microfluidics or bioreactors, pushing culturing times up to 7–10 days (Holton et al., 2017; Aref et al., 2018).

Overall, PDMEs offer a highly representative platform to be used to predict response to clinical therapy when taking the tumor microenvironment into account. Some examples include when it has been used to select chemotherapy in untreated, advanced or metastatic non-small cell lung cancer (NSCLC) (Nagourney et al., 2012). This strategy allowed a 2-fold improvement over historical control of 30%. PDMEs were also used successfully in prostate (Centenera et al., 2018; Risbridger et al., 2018) and breast cancer (Carranza-Torres et al., 2015), with 100% survival after 96 h. In some studies, the PDMEs were not simply immersed in media but sometimes placed on substrates such as titanium or stainless grids, or gelatin sponges (Geller et al., 1997; Centenera et al., 2012, 2013, 2018; Schiewer et al., 2012; Risbridger et al., 2018). This prevented cell outgrowth from tumor tissues, which may often occur, as seen in prostate cancer PDMEs for example, maintaining viability for up to one week of culture (Centenera et al., 2013). Importantly, unlike PDXs and PDOs, PDMEs still maintain stromal and immune cells, which enable drug screening in immuno-oncology, such as the immune checkpoint blockade (ICB), which is impossible for any other 3D approach that lack an immune compartment. This has been heavily investigated using microfluidic devices (Aref et al., 2018; Jenkins et al., 2018; Moore et al., 2018; Wang et al., 2018a).

The most significant disadvantage in PDMEs is the poor control of sizes used for experiments. Often, there is little control over dimensions and pieces are grossly cut. Even when the fragments are sieved, fractions still include large variations (with often more than 2-fold size differences) resulting in increased degrees of heterogeneity, which unnecessarily increases variability in drug responses, in a context where it should be kept to a minimum. In this respect, organotypic slices represent a much more reproducible way to culture explants for drug testing purposes.

##### Organotypic tissue slices (OTS)

OTS are thin sections prepared from whole tumor tissue, which are cultured either as floating pieces or on a supporting structure. Currently, OTS are best at taking into consideration intratumoral heterogeneity and the tumor-stromal interactions of *in vivo* tumors (Meijer et al., 2017). OTS are able to retain the complexity of the tissue environment, unlike the dissociation of tissue required for organoid culture, however only for a short amount of time. OTS contain the native cells that support heterogeneous phenotypes. Although OTS have many advantages, they have become less utilized in modern research. This is mostly due to a low number of samples that can be generated from biopsy tissue, their inability to be passaged, and the limited timeframe available to study the samples during culture.

OTS have mainly been used for the study of chemotherapeutic response to various cancer tissues (Holliday et al., 2013; Merz et al., 2013; Gerlach et al., 2014; Koerfer et al., 2016; Naipal et al., 2016). Automated slicing, via tissue slicers and vibratomes, has enabled the maintainance of tissue integrity and minimal handling of the tissue pieces, ensuring higher viability (Krumdieck et al., 1980). The thickness of slices needs to allow for appropriate media perfusion but also maintain tissue architecture, most often this occurs around 300 μm (Risbridger et al., 2018). Some reports suggested that smaller tumors may need to be embedded in agarose gel prior to sectioning (Davies et al., 2015). Tumor texture also relates to its ease of slicing as soft, mucinous or fibrous tumor sections could not be sliced into sections <500 μm (Holliday et al., 2013; Gerlach et al., 2014; Naipal et al., 2016). Automated slicing has been used extensively in the preparation of OTS for NSCLC (Vaira et al., 2010; Davies et al., 2015), brain (van der Kuip et al., 2006; Holliday et al., 2013; Merz et al., 2013; Carranza-Torres et al., 2015; Davies et al., 2015; Naipal et al., 2016), colon (Vaira et al., 2010), prostate (Hällström et al., 2007; Vaira et al., 2010; Zhang et al., 2018b), HNSCC (Gerlach et al., 2014), and pancreatic tumor tissues (Lim et al., 2018; Misra et al., 2019).

OTS can be cultured in various ways, most often as floating in medium or supported by a membrane. The use of a supporting structure has been a key feature of OTS cultures for some time. In earlier publications this was served by titanium or stainless steel grids (Parrish et al., 2002; Hällström et al., 2007), and in more recent publications, by the Millipore cell culture inserts (Vaira et al., 2010; Merz et al., 2013; Gerlach et al., 2014; Koerfer et al., 2016; Misra et al., 2019). Some research groups also use gelatin sponges to support OTS cultures (Papini et al., 2007), to prevent an unrelated inflammatory response at the surface of each slice. As a comparison, Davies et al. (2015) maintained cultures for 72 h either floating in medium, or on a Millipore cell culture insert, before fixation and histological sectioning. OTS as floating pieces displayed alterations in their stress pathways and also a loss of tissue integrity, while these changes were not apparent for slices cultured on membranes. A local microenvironment was established at the point where the air and filter met, mimicking oxygen gradients as present in tumors *in situ*. For static floating cultures, it is suggested that a lack of oxygen and nutrient perfusion is most likely the reasoning behind short tissue viability (Davies et al., 2015). Floating cultures are often sustained for a longer time by using a rotating device to ensure perfusion (Pretlow et al., 1995; Naipal et al., 2016). Well-defined media supplementation (Naipal et al., 2016), or the use of autologous serum (Majumder et al., 2015), can also lead to longer culture durations or improved clinical relatability. Autologous serum, while highly relevant to interpatient heterogeneity, also contains a degree of variability arising from the patients' past clinical history (Majumder et al., 2015). The longest OTS culture durations we found to be published was by Merz et al. (2013) who prepared primary glioblastoma tissue slices to a thickness of 350 μm, on Millipore cell culture inserts. Twelve patient samples were able to maintain the original tumor structure and phenotype for a minimum of 16 days.

When incorporated into preclinical studies, OTS enable the quantitative evaluation of clinically relevant endpoints. Undoubtedly, the ability to visualize the effect of treatments on the tissue as an entire structure (undigested), including native tumor heterogeneity, provides a broader overview than with those techniques involving tissue digestion and reformation, albeit for a short duration. Additionally, the opportunity to culture tumor tissue alongside adjacent normal tissue allows for the testing of therapeutics that target malignant cells while not affecting the healthy surrounding cells. In future, to fully leverage the value of OTS, users may need to consider high-throughput live spinning disc and light-sheet confocal microscopy, which, when performed on entire OTS, will provide a significant advantage compared to static analysis. Such a technique will enable to observe temporal responses to drug treatments according to various spatial zones. This approach may report live cellular mechanisms according to drug responses to hopefully an even greater degree than seen with intravital microscopy on animals.

### Scaffold-Based Approaches

Scaffolds-based systems provide a toolkit where both tumor and stroma-derived materials can be cultured. Using natural or synthetic matrices with tailorable chemical and physical cues, the influence of various microenvironmental factors may be studied. While innovative and more relevant strategies are constantly being reported, Matrigel is still today the gold standard in 3D cell culture of patient-derived materials, despite lack of tenability, and despite being derived from a mouse tumor ECM. Hereafter, we will focus on all other scaffold-based alternatives, with a focus on synthetic/semi-synthetic hydrogels and tissue-engineered scaffolds, or combination of the above (Table 2).

**Table 2:** Overview of scaffold-based tumor models from patient-derived materials.

**Main cancer type**	**Purpose and application**	**Patient numbers**	**Method**	**Stromal cell components**	**Maximum culture time**	**References**
**HYDROGEL-BASED**						
Acute myeloid leukemia	Drug response; biological studies	3	PEG-Heparin hydrogels	HUVECs and MSCs	14 days	Bray et al., 2017
Appendiceal cancer	Drug response; immunotherapy testing	12	HA-Collagen hydrogels	Lymph node cells	11 days	Votanopoulos et al., 2018
Brain cancer and kidney cancer	Drug response in parallel with the clinic	5	VersaGel	–	15 days	Hribar et al., 2019
Breast cancer	Biological studies into matrix deposition	Not stated	Gelatin porous microbeads cultured in a spinning flask	CAFs and normal fibroblasts	12 days	Brancato et al., 2017
Breast cancer and brain metastasis	Biological studies into cancer cell migration	15	Cells were pre-grown in 2D, cell aggregation using nucleo-pore filters membrane; PEG-HA-Collagen hydrogels	CAFs from normal, primary, and brain metastatic samples	4 weeks	Chung et al., 2017
Breast cancer	Biological studies	Not stated	Gelatin cryogels (GelMA)	CAFs	3 days	Zhang et al., 2017
Liver cancer	Drug response; biological studies	16 PDX tumor samples	MA-HPC sponges	–	20 days	Fong et al., 2018
Lung cancer	Drug response	2	Collagen-HA hydrogels	–	5 weeks	Mazzocchi et al., 2019
Multiple Myeloma	Biological studies	Not stated	Fibrinogen gels, PLGA microspheres, Aligimatrix, and Matrigel	HUVECs and stromal cells from MM patients	7 days	de la Puente et al., 2015
Prostate cancer	Drug response	2 PDX tumor samples	PEG-HA hydrogels	–	14 days	Fong et al., 2014
**TISSUE-ENGINEERED SCAFFOLDS**						
Breast cancer	Drug response; biological studies	4	PCL porous scaffold	Immortalized CAFs were pre-cultured on PCL scaffolds and then decellularized	10 days	Nayak et al., 2019
Prostate cancer	Biological studies	3	PCL scaffolds and Matrigel	Osteoblasts	3 weeks	Shokoohmand et al., 2019
Prostate cancer	Drug response; biological studies	2 PDX tumor samples	PCL scaffolds	Osteoblasts	30 days	Paindelli et al., 2019
Prostate cancer	Biological studies in ECM remodeling	14 matched fibroblast samples	PCL scaffolds	CAFs and mast cells	2 days of co-culture	Pereira et al., 2019

*CAFs, cancer-associated fibroblasts; ECM, extracellular matrix; HA, hyaluronan; HUVEC, human umbilical vein endothelial cells; PEG, polyethylene glycol; PCL, polycaprolactone; PDX, patient-derived xenograft; PLGA, poly(lactic-co-glycolic acid); MA-HPC, methacrylate-hydroxypropylcellulose; MSCs, mesenchymal stem cells*.

#### Hydrogels and Tissue-Engineered Scaffolds

##### Hydrogels

PDOs represent a significant improvement in the biomimetic culture of primary tumor cells. Yet one issue lies in the lack of malleable surrounding matrix that prohibits spatial control and controlled additions of multiple cell layers (Fong et al., 2016b). Semi-synthetic and synthetic materials offer inertness and therefore an ability for cells to deposit their own ECM rather than being cued to develop a specific phenotype or morphology. This means that decreased biomaterial heterogeneity is achievable when using synthetic materials, while Matrigel compounds patient heterogeneity with its own interscaffold and interbatch heterogeneity (Postovit, 2016). The state-of-the-art in 3D bioengineered models, pre-dominantly polyethylene glycol (PEG)-derived with a glycoprotein component, allows for control over added ECM proteins while supporting the development of natural matrix deposition. These approaches are being constantly improved and have resulted in the generation of novel materials, however applications toward primary patient-derived tumor cell cultures has been more rare (Li and Kumacheva, 2018).

**Figure 4:**
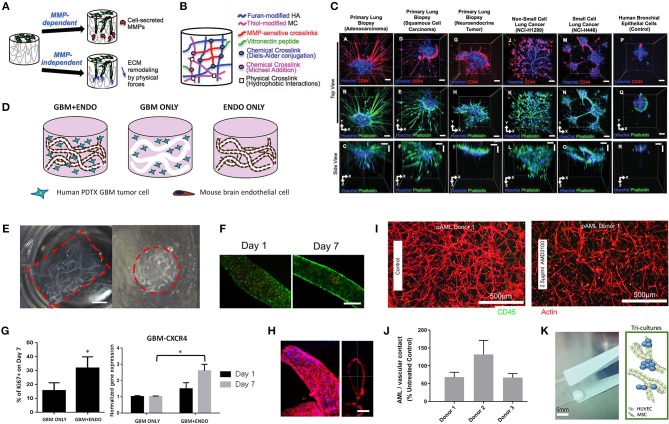
Hydrogel culture approaches. **(A–C)** Rationally designed 3D hydrogels model normal and malignant lung tissue. **(A)** Schematic of potential cell invasion mechanisms. **(B)** Schematic of the composition of biomimetic stimuli-responsive 3D hyaluronan (HA) hydrogels. **(C)** Lung cancer cells express CD44 and show varying invasiveness into 3D HA hydrogels. (A–I) Primary cells isolated and cultured from three separate lung carcinoma biopsies identified as (A–C) adenocarcinoma, (D–F) squamous cell carcinoma, and (G–I) neuroendocrine tumor. (J–L) Non-small cell lung cancer and (M–O) small cell lung cancer cells. (P–R) Healthy human bronchial epithelial control cells do not invade into 3D hydrogels. (A,D,G,J,M) Lung cancer cells express CD44, while (P) healthy bronchial epithelial cells do not. (D–H) Combination approach to mimicking glioblastoma using PDX derived cell lines. **(D)** Schematic of hydrogel/microfiber structure and cellular combinations. **(E)** Cross-sectional views of encapsulated alginate microfibers in 3D hydrogels. Left = side view. Right = top view. Red = hydrogel edge. Scale bar = 2 mm. **(F)** Live (green) and dead (red) staining of endothelial cells after 7 days in cultures. **(G)** Co-culture with mouse endothelial cells increased glioblastoma proliferation via Ki67 staining (*n* = 150) (left), and increased expression of CXCR4 in glioblastoma tumor cells (right). *p < 0.05. **(H)** Confocal image demonstrating CD31 expression from HUVECs within alginate microfibres. **(I–K)** 3D culture of acute myeloid leukemia using starPEG-heparin hydrogels. **(I)** AML cells from a patient with AML untreated (left) or treated with 2.5 μg/mL AMD3100 (right), co-cultured with HUVECs and MSCs. **(J)** Percentage of AML contact with HUVECs and MSCs decreased after AMD3100 treatment in two out of three donors, compared with the untreated control sample. Means ± SD (variability within experiment, *n* = 1). **(K)** A biohybrid starPEG-heparin hydrogel for the culture of AML mono-cultures and tri-cultures with HUVECs and bone marrow-derived MSCs. Scale bar = 5 mm. **(A–K)** reproduced with permission from Tam et al. (2018), Wang et al. (2019), and Bray et al. (2017), respectively.

Hribar et al. (2019) demonstrated the culture of glioblastoma and renal cell carcinoma within a photocrosslinkable hydrogel called VersaGel, a growth factor free platform with integrin binding sites and MMP degradability. VersaGel was demonstrated to support the growth of dissociated cells and tumor fragments from PDX samples or patient tissue. Prior culture in ULA flasks promoted spheroid formation before being plated and into VersaGel. Gels incubated in conditioned media from the original ULA spheroid cultures resulted in an invasive phenotype of the renal cancer PDX tissue while fresh media resulted in more tightly packed spheroids. Five patient samples of glioblastoma were also cultured within the VersaGel and exposed to temozolomide, a first-line chemotherapy treatment for glioblastoma. The response was compared with Matrigel, finding that while VersaGel therapeutic response correlated with all five patient clinical responses, Matrigel culture correlated with only three out of the five patients. A combinatory hydrogel approach was used by others (Mazzocchi et al., 2019) to culture two lung cancer samples, isolated from pleural effusion, the excess fluid found between the pleura and lungs. The hydrogels were composed of methacrylated collagen I and thiolated HA, using UV polymerization. Cultures were maintained for 6 weeks and preserved the heterogeneity of the cell populations, and chemotherapeutic treatment was less effective on gels compared to 2D cultures. In another combined hydrogel approach, HA-collagen hydrogel models were created from 12 patients with appendiceal cancer (Votanopoulos et al., 2018). In some cultures, the researchers added cells derived from the patient's lymph nodes in addition to the tumor samples from the same patient to “immune enhance” the culture. From the 12 patients, 75% of the cultures could be established. The high-grade tumors demonstrated tissue-like structures within the hydrogels, whereas the low-grade tumors showed more spread out cells/organoids. Interestingly, the low-grade tumors did not respond to chemotherapy, while the high-grade tumors had a variable response. In the tumors co-cultured with lymph node cells, increased mitochondrial metabolic activity was demonstrated in organoids treated with immunotherapeutics, 24 h after first exposure. However, 96 h after exposure, decreased mitochondrial metabolism was seen in the treatment groups. These interactions with immune cells are a key part of recapitulating the tumor microenvironment, especially in immunotherapy research. Tam et al. (2018) developed a metastatic lung cancer model using a biomimetic hydrogel platform also containing HA (Figures 4A–C). To mimic the viscoelastic features of lung tissue, methylcellulose was added to the 3D model. MMP-mediated cell migration and invasion was accounted for by including collagen-I-derived peptide crosslinkers that could be enzymatically degraded by cell-secreted MMPs. The researchers modified their culture platform to develop a 384-well format in order to enable high-throughput drug screening, a key priority for the future of patient-specific *ex vivo* models.

A fusion of PDX samples and tissue engineering was performed by Fong et al. (2014). Immediately after the PDX prostate tumor dissociation, the cell pellets were resuspended in HA-PEG hydrogels, where the PEG component had been modified with the tripeptide Arg-Gly-Asp (RGD) and MMP-cleavable sequences. In some cases, the PDX samples were co-encapsulated with MC3T3-E1 osteoblastic cells. In the model, the osteoblastic cells spread over time, while the PDX prostate cancer samples remained as aggregates. The co-culture resulted in higher proliferation than the individual mono-cultures, demonstrating effective cell-cell signaling within the model. Moreover, this study demonstrated strong structural and phenotypic similarities between the original patient tumor, the murine PDX model and the *in vitro* hydrogel model. Fong et al. later published a novel microporous hydrogel sponge derived from hydroxypropylcellulose methacrylate to culture 16 liver cancer PDX samples. Of those 16 samples, two were not viable within the system, suggesting tumor diversity amongst the samples (Fong et al., 2018). In our own work, we have previously used semi-synthetic PEG-heparin hydrogels for the culture of patient-derived samples (Chwalek et al., 2014; Bray et al., 2015, 2018; Taubenberger et al., 2016).

Most recently, we published a study investigating the growth of human acute myeloid leukemia (AML) cells within these hydrogels and treated them with first-line chemotherapy (Figures 4I–K) (Bray et al., 2017). Cell lines and primary AML cells derived from the peripheral blood of three patients displayed a tendency to grow along the vascular network derived from human umbilical vein endothelial cells (HUVECs) and MSCs. However, while the cell lines proliferated throughout the culture, the primary AML cells were maintained but not propagated. Cultures were maintained for 7 days before chemotherapy treatment, with varied results between donors. A study from de la Puente et al. (2015) developed a multi-cellular culture of multiple myeloma cells, stromal cells (derived from multiple myeloma patients), and HUVECs using fibrinogen hydrogels. The fibrinogen was compared with poly(lactic-co-glycolic acid) (PLGA) microspheres, AlgiMatrix, and Matrigel. Using the fibrinogen model, the authors found that the co-culture of patient-derived multiple myeloma (MM) cells with stromal cells resulted in increased proliferation of MM cells, this further increased when the endothelial cells were also added, showing the importance of adding supporting cell types to tumor microenvironment models. This is an interesting finding, as MM cells are notoriously difficult to cultivate using 2D conditions, and most often do not grow at all *ex vivo*. When looking at other matrix types, PLGA microspheres did not support patient-derived MM proliferation, AlgiMatrix, and Matrigel supported a small amount of MM proliferation, while the fibrinogen scaffolds supported a 250% increase in the proliferation of three patient MM samples, perhaps due to it being a natural component of blood and marrow plasma. These scaffolds also created oxygen gradients, whereby higher levels of hypoxia-inducible factor 1-alpha (HIF1α) and pimonidazole were found in the lower layer of the scaffold, while drug penetration was reversely correlated with scaffold depth.

In the glioblastoma research field, progress in 3D tumor modeling has occurred using synthetic PEG hydrogels combined with alginate microfibers (Figures 4D–H) (Wang et al., 2019). The researchers utilized a patient-derived adult glioblastoma xenograft cell line (D-270 MG) combined with a mouse brain microvascular endothelial cell line. The tumor cells were resuspended in a PEG-HA hydrogel precursor solution with MMP cleavable and RGD peptides. These tumor cells were then either co-cultured with acellular alginate microfibers (formed via photocrosslinking) or with the endothelial cell line pre-suspended in the alginate solution. These endothelial monolayers were well-formed in monoculture, however were disorganized and cells became rounded in co-cultures with tumor cells. After 14 days, glioblastoma tumors near the endothelial channels were more spherical, while those tumors in monoculture were more migratory. However, the use of mouse endothelial cells with human glioblastoma cells will not provide a realistic response. It is also worthwhile to note that the microfiber channels created did not allow for perfusion or flow. Nonetheless, the spatial organization of the capillary structures with the combination of hydrogel materials allows for the reconstruction of a useful *in vitro* model. Future studies with perfusable capillaries would enable analysis from the perspective of nutrient and oxygen delivery to the tumor.

The recapitulation of the tumor microenvironment goes beyond the culture of tumor cells. A recent study used gelatin porous microbeads to create either microtissue constructs or produce spheroids (Brancato et al., 2017). CAFs or normal fibroblasts were loaded together with the microbeads into a spinning flask or seeded into round bottom, non-treated 96-well plates in methylcellulose solution and maintained for up to 12 days. The biophysical properties of the methylcellulose models were relatively similar between CAFs and normal fibroblasts, however marked differences were apparent when cultured on the microbeads, highlighting the importance of structural considerations in model development. There was an increase in matrix deposition by the CAFs, a higher proliferation rate and a higher stiffness of microtissue compared to that created by the normal fibroblasts. Additionally, cryogels can be created by forming hydrogels below the melting point of a solvent. Cryogels formed from PEG-heparin have been reported by our group (Bray et al., 2018), where they were used to create a bone microenvironment using mineralized primary human osteoblasts for co-culture with breast cancer cells lines. Another study utilized a gelatin-based cryogel modified with methacrylate groups (GelMA) (Zhang et al., 2017). The authors used the cryogels to create a tumor stromal microenvironment using CAFs derived from breast cancer patients, showing increased cancer cell migration compared to their mono-cultures. CAFs were also utilized by others (Chung et al., 2017) to study their effects on breast cancer primary and brain metastatic cell migration. Using a PEG-HA-Collagen hydrogel, they found that significantly higher numbers of patient-derived tumor cells migrated toward CAFs derived from brain metastatic samples, supporting the theory of the pre-metastatic niche and highlighting the effectiveness of such cytokine gradients in cancer cell attraction.

The creation of these microenvironmental changes that a local tissue undergoes during malignant transformation is an important and often overlooked aspect of tumor engineering, which needs to be incorporated in cancer modeling. Nonetheless, it should be noted that despite what a researcher may gain in reproducibility using semi-synthetic and synthetic hydrogel matrices, including decreased material interference, there is a loss of rich ECM components that are found in Matrigel. Therefore, it must be ensured that these synthetic models are fully characterized to determine that interpatient and intertumoral heterogeneities are maintained similarly to Matrigel-based models. The most suitable ECM recapitulation *in vitro* depends on the tissue of choice. Collagen would be the most significant component of many tumor tissues, including breast, prostate and colorectal regions. Aside from collagens, ECM contributions also arise from proteoglycans, laminins, and fibronectin, all significant in the context of tumor progression. Applications for heparin and HA-derived hydrogels were described above, however further work could be performed to include other glycosaminoglycans such as chondroitin sulfate in the engineering of semi-synthetic hydrogel materials. In the tissue engineering space, it has also been possible to integrate peptide motifs into synthetic materials to mimic collagen I (GFOGER), laminin-111 (IKVAV), and fibronectin (RGD) for ECM mimicry (Taubenberger et al., 2016), however these were not tested using patient-derived tissues. In some cases, the incorporation of ECM components into synthetic hydrogels may not be necessary if included supporting cells are able to deposit their own matrix readily.

##### Tissue-engineered scaffolds

Scaffolds applied to the engineering of tumor microenvironments are based on natural or synthetic polymers which offer a high degree of tailorability for a targeted microenvironment. Constructs made from natural polymers (alginate, chitosan) offer low toxicity with components similar to natural ECM, yet have weak mechanical properties and limited options for fine-tuning of degradability or chemistry. Synthetic constructs can be made from medical-grade polymers (polycaprolactone (PCL) and PLGA-based) that offer more reproducibility and tailoring options in terms of chemical and mechanical properties.

**Figure 5:**
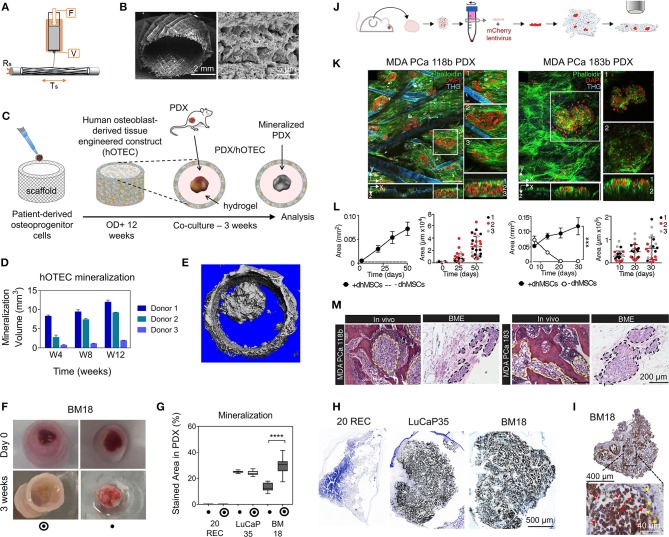
Tissue-engineered model approaches combining scaffolds, patient-derived materials, and primary cells. **(A–I)** prostate cancer (PCa) PDX osteomimicry when co-cultured with a patient-derived mineralized microtissue scaffold. **(A)** Schematic of melt electrowriting of medical grade polycaprolactone (mPCL) into a porous tubular microfiber scaffold. **(B)** SEM images of mPCL scaffold after calcium phosphate treatment to induce osteogenic properties. **(C)** Scaffold seeding with patient-derived osteoprogenitor cells, cultured for 12 weeks under osteogenic differentiation leading to a human osteoblast-derived tissue engineered construct (hOTEC), followed by co-culture with PDX for 3 weeks. **(D)** Mineralization differences in hOTECs according to patients. **(E)** Micro-computed tomography image of bone metastasis-derived PCa PDX (BM18) in co-culture with hOTEC shows high mineralization of both hOTEC and PDX mass after 3 weeks (Mean ± SE). **(F)** Photographs of BM18 PDX, cultured either alone or co-cultured with hOTEC at day 0 and after 3 weeks of culture. **(G)** Mineralization quantification from von Kossa staining inside PDX, shows that BM18 became more mineralized than lymph node-derived PCa PDX (LuCaP35) and endometrial cancer metastasis-derived PDX (20REC), in the presence of hOTEC. **(H)** Von Kossa staining shows strong mineralization in PCa PDXs (BM18 and LuCaP35) but no mineralization in the control endometrial PDX (20REC). **(I)** NuMA staining in BM18 PDX shows a majority of human cells (>75%, red arrows = human, yellow arrows = mouse). **(J–M)** PCa PDX-derived cells growth in a bone mimetic environment (BME). **(J)** Process schematic; PCa PDX (MDA PCa 118b and 183) were extracted from mice, dissociated in single cells, and transfected with mCherry lentivirus prior to co-culture on an osteoblast-derived microtissue made from melt electrowritten mPCL porous scaffolds populated with immortalized human MSCs differentiated into osteoblasts (dhMSCs) for 30 days prior to co-culture. **(K)** Multiphoton microscopy of tumor cells co-cultured with dhMSC scaffolds. **(L)** Growth areas of tumor cells on scaffolds ± dhMSCs shows no survival without dhMSCs and increase in the presence of dhMSCs. **(M)** Histology of MDA PCa 118b and MDA PCa 183 in bone and BME. Yellow and black dashed lines outline the tumor areas. **(A–M)** reproduced with permission from Shokoohmand et al. (2019) and Paindelli et al. (2019), respectively. ****P < 0.0001.

While hydrogels are more relevant to mimic soft tumors, hard scaffolds may suit better tumor sites with higher stiffness, such as hard bone, which stiffness range from 2 to 10 GPa (Qiao and Tang, 2018). Medical grade PCL (mPCL) has been widely used for bone tissue engineering applications due to suitable viscoelastic properties and low melting temperature enabling easy processing into various scaffold architectures (Woodruff and Hutmacher, 2010). While this has been heavily investigated for *in vivo* applications, mPCL is now also used in *in vitro* cancer models, mostly printed as microfiber 3D architectures enabling seeding and culture of bone cells. In our work (Shokoohmand et al., 2016, 2019; Bock et al., 2019), we have used melt electrospinning combined with additive manufacturing (“melt electrowriting”) to print mPCL microfibers into linear or tubular porous scaffolds populated with primary osteoprogenitors isolated from human bone tissue (Figures 5A–C). By coating the fibers with calcium phosphate and using osteogenic differentiation media, the resulting osteoblast-derived microtissues contained osteoblastic and osteocytic cells with abundant key ECM deposition. The patient-derived microtissues were used as an *in vitro* mineralized model platform to study prostate cancer growth in bone, by co-culturing cancer lines (Bock et al., 2019) and PDXs (Shokoohmand et al., 2019). In the PDX study (Figures 5D–I), prostate cancer PDX models were used; from lymph node metastasis (LuCaP35) and bone metastasis (BM18). PDXs were supported by Matrigel in the center of the tubular osteoblast-derived microtissues and cultured for 3 weeks. The co-culture generated bone mimicry of both PDXs at the gene, protein and mineralization levels. Interestingly, while the PDX co-cultures were done with microtissues made from the osteoprogenitor cells from only one donor, the study reported that the microtissues were initially made with cells from three different donors. While reproducible for cells from one patient, the bone microtissues showed donor heterogeneity, with one patient displaying poor mineralization of the construct, which was explained by a severely obese BMI. These results spoke of the importance of using primary cells in 3D culture system models to ensure tissue engineering of a relevant patient-specific context. In future, Matrigel could be replaced by attractive synthetic options such GelMA, to avoid a murine component in this otherwise fully human model. Using a similar scaffold design as in Bock et al. (2019), melt mPCL electrowritten scaffolds were used to create a osteoblast-like microenvironment, although using differentiated immortalized human MSCs (Figures 5J–M). Two patient-derived PDX samples were dissociated and cultured on top of the mineralized microtissue up to 50 days for 1 patient and 30 days for the other. PDX cells from neither donor survived when the construct was stroma-free. This indicates the need for stromal context to support longer term cultures of patient-derived components (Paindelli et al., 2019).

Melt electrowritten mPCL scaffolds were also used to culture prostate CAFs and normal fibroblasts and facilitate ECM deposition to create a microtissue construct with stromal context (Pereira et al., 2019). It was reported that BPH-1 benign prostate hyperplasia epithelial cells altered their sphericity, orientation and cell length when cultured on the CAF microtissues when compared with normal prostate fibroblasts. Similarly, Nayak et al. (2019) utilized a PCL porous scaffold, created via a salt leaching technique, to culture two patient-derived breast cancer specimens with an ECM matrix deposited by immortalized CAFs. In this instance, the PCL scaffold with CAFs was decellularized after ECM deposition. The presence of the CAF ECM increased the breast cancer epithelial cells viability and cell-matrix interactions when compared with bare PCL scaffolds. The drug response of the breast cancer cells varied between patients, indicating maintained interpatient heterogeneity (Pereira et al., 2019).

#### Additive Biomanufacturing/3D Bioprinting

Most scaffold-based or scaffold-free approaches to design 3D *in vitro* tumor models present limitations such as limited control over cellular and matrix patterning, limited simultaneous deposition of multiple cell populations and/or ECM types, low throughput, manual production, and batch-to-batch variability. Additive biomanufacturing, or bioprinting, is a versatile alternative that allows the reproducible manufacturing of complex, spatially-defined 3D biostructures (Li et al., 2018a). Traditionally, 3D bioprinting can be achieved by extrusion, inkjet or laser assisted (Albritton and Miller, 2017; Tsai et al., 2017). Comprising multiple cell types or tissues, bioprinted multicellular models can more truthfully recreate specific microenvironments for modeling of both normal and diseased tissues (Ma et al., 2018). Most recently, this versatile technology has enabled the generation of more reproducible and complex *in vitro* cancer models (Knowlton et al., 2015; Zhang et al., 2016; Albritton and Miller, 2017; Ma et al., 2018), by simultaneously printing multicellular cancer and stromal compartments. Initially though and still currently, bioprinted systems are heavily composed of cell lines. Yet, a small portion of studies are starting to display patient-specific components, found either in the cancer (Langer et al., 2019) (rarer) or stromal (Zhou et al., 2016; Wang et al., 2018b) compartments and has enabled the assessment of patient specificity and microenvironment heterogeneity better than previously simplified 3D systems.

**Figure 6:**
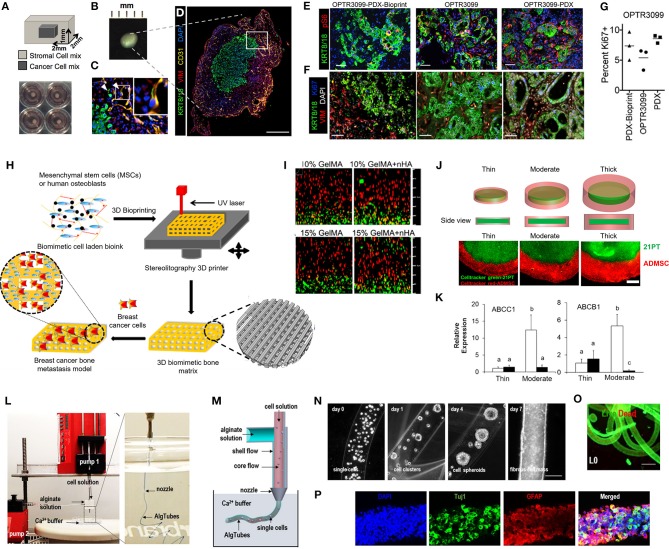
Bioprinting with patient-derived materials and primary cells. **(A–G)** Bioprinted tissues from pancreatic patient-derived xenograft PDX-derived materials surrounding by a mixture of primary stellate cells (PSCs) and human umbilical vein endothelial cells (HUVECs) and comparison with original tissue. **(A)** Schematic of bioprint structure and photographs of bioprints in normal tissue culture plates. **(B)** Photograph of individual bioprint. **(C,D)** Low and high magnification of immunofluorescence (IF) images of bioprints from PDX-derived cell line after 7 days in culture, showing KRT8/18 (cancer cells) in green, vimentin (VIM, stroma) in red and CD31 (vasculature) in yellow, and DAPI (nuclei) in blue. **(E)** IF for KRT8/18 (green), pS6 (red), and DAPI (blue) of OPTR3099-PDX-Bioprint tissue (PSCs and HUVECs in the stromal compartment with disassociated PDX tumor tissue generated from OPTR3099 in the cancer compartment), primary patient tissue from OPTR3099, and PDX tumor tissue generated from OPTR3099 (OPTR3099-PDX). **(F)** Similar tissue to **(E)**, except that IF is for KRT8/18 (green), VIM (red), Ki67 (blue), and DAPI (gray). **(G)** Ki67+ quantification of the percentage of Ki67+/KRT8/18+ dual positive cells shown in **(F)**, *n* = 3 random fields of view, *N* = 1 PDX bioprint. **(H,I)** Bioprinted breast cancer bone metastasis model. **(H)** Schematic of primary mesenchymal or osteoblast cell line-laden GelMa-based 3D bioprint from stereolithography and further co-culture with breast cancer cell lines. Four groups were used, containing either 10 or 15% GelMA ± nanohydroxyapatite powder (nHA). Insert shows CAD model of the 3D matrix (gray) and 3D surface plot of the bioprinted matrix (colored image). **(I)** Confocal micrographs of mesenchymal stem cells (MSCs)-laden 3D bioprints 1 day post printing (cross-sectional views) for each bioprint group. Live (green) and dead (red) cells. Over 75% of cells were dead after bioprinting. **(J,K)** Bioprinted primary breast cancer model. **(J)** 21PT breast cancer line cells were first bioprinted in a photocrosslinkable gel followed by printing hydrogels of primary adipose derived MSCs (ADMSCs) around the cancer cell gel, with various thicknesses. ADMSCs in the edge region were labeled by cell tracker red, and 21PT in the middle region were labeled by cell tracker green (fluorescence images). **(K)** qPCR analysis of adenosine triphosphate (ATP)-binding cassette transporter gene expression of the bioprinted constructs with and without the lysyl oxidase (LOX) inhibitor, *n* = 3. **(L–P)** Glioblastoma tumor-initiating cells (TICs) culture in alginate hydrogel tubes (AlgTubes). **(L)** Images of extrusion system. **(M)** Schematic of AlgTubes production. **(N)** TIC growth in AlgTubes. Scale bar: 200 μm. **(O)** Live/dead staining of day 7 cells in AlgTubes. Scale bar: 400 μm. **(P)** *In vitro* differentiation of TICs after 10 passages. Scale bar: 100 μm. **(A–P)** reproduced with permission from Zhou et al. (2016), Wang et al. (2018b), Langer et al. (2019) and Li et al. (2018b), respectively.

In a 2019 key study in the field (Figures 6A–G), Langer et al. used a Organovo's Novogen MMX bioprinter platform to print millimeter-size scaffold-free structures composed of a cancer core surrounded by patient-derived stromal cell types (Figures 6A–D) (Langer et al., 2019). A pancreatic cancer PDX cell line (OPTR3099C) and two primary patient tumor (OPTR) tissues were used for the inner core. The stromal component, comprising the outer core, was half primary with a composition of HUVECs mixed with primary pancreatic stellate cells (PSCs). The hydrogel was made of an alginate-containing gelatin hydrogel, and designed to dissolve after 48 h in culture at 37°C. The OPTR/stromal bioprints recapitulated the morphological structures of the corresponding PDX analog and primary tissue. Signal heterogeneity was also recapitulated by assessing pS6 staining, a readout of mTOR signaling. However, the tumor tissue and original PDX showed clear pS6 staining not only in the cancer cells, but also in the surrounding stroma, but was not seen in the bioprints (Figure 6E). Heterogeneous staining within cancer cell areas was similar between bioprints, PDX, and primary tissue. Overall, all bioprinted tumor models displayed low (<10%) levels of proliferative cells (assessed by Ki67+, Figures 6F,G), similar to native tissues. While viability was not assessed, the addition of various cells or different drug treatments showed quantifiable effects more akin to the clinical scenario. For example, the use of PSCs in the stromal mixture showed a more reactive ECM-rich tumor microenvironment, and the efficacy of drugs such as dactolisib (a PI3K inhibitor) was reduced with the addition of fibroblast conditioned-media. This was anticipated as it has been suggested in the past that paracrine factors from fibroblasts may contribute to dactolisib therapeutic resistance, which was recapitulated in the bioprints. In summary, this “vitrine” study suggested the possibility to capture heterogeneity in therapeutic response, migration, and signaling using a combination of patient-derived materials and supportive cell lines (Langer et al., 2019).

Other bioprinted systems have used cancer cell lines but have used bioprinted stromal compartments with primary cells. This strategy is relevant when the focus is the study of cancer/stroma interactions. This was adopted in a breast cancer bone metastasis model with bone marrow (BM)-MSCs printed in various photocurable GelMA bioinks (Figure 6H) (Zhou et al., 2016). In co-culture, the BM-MSCs increased the proliferation of cancer cells and vascular endothelial growth factor expression, while reciprocal effects involved reduction of alkaline phosphatase activity in BM-MSCs. Similar results were seen in both MSCs and an osteoblast cell line (hFOB 1.19). However, when comparing the viability between the osteoblast cell line bioprint vs. primary BM-MSCs, the latter were significantly damaged by the process, with >75% of dead cells upon bioprinting in all GelMA bioink variants (Figure 6I), which was less for the osteoblast cell line (Zhou et al., 2016). This result suggested that primary cells may be more sensitive than cell lines for bioprinting, warranting a thorough viability assessment for bioprinted models.

In another breast cancer study (Figures 6J,K), dual hydrogel-based bioinks were extruded to have a cancer cell line core and a stroma shell made of primary adipose-derived MSCs (ADMSCs) with various layer sizes (Wang et al., 2018b). Both compartments were printed successively, and with different biomaterials properties. Overall, the bioinks contained HA and gelatin, that were methacrylated or not. A softer mixture (leading to ~400 Pa) was used for the cancer cells while a higher concentration of the components (leading to ~1,000 Pa) was used for the ADMSC gels to promote cell spreading. Overall, HA and gelatin, without modification, were used to increase the viscosity and printability and maintain the softness of the bioprinted constructs for cell migration. Heterogeneity here was addressed from the structural point of view by varying the thickness of the stromal layer (thin: 0.4, moderate: 0.8, thick: 1.2 mm) mimicking obesity status. After culturing for 21 days, doxorubicin (DOX) and LOX inhibitor responses were assessed for 3 days. Apoptosis rates were lower for the moderate and thick ADMSC layers. Interestingly, LOX, which drives the cross-linking of collagen and elastin and is negatively associated to breast cancer progression, was expressed regardless of changes in the ADMSC layer thickness or DOX administration. However, qPCR results showed that a thicker ADMSC layer upregulated multidrug resistance-related genes such as ABCC1, ABCB1, and ABCG1, which were accordingly reduced in the presence of the LOX inhibitor but only significantly in the moderate ADMSC layer (Figure 6K). Finally, the researchers showed that ADMSCs rather than hypoxia (as measured by HIF1α) was the major contributor to drug resistance (Wang et al., 2018b).

In a recent glioblastoma study, dissociated tumor-initiating cells (TICs) mixed with 2% HA were extruded in macroscopic alginate tubes (400 μm, Figures 6L,M) (Li et al., 2018b). The cells filled the tubes within 7 days (Figure 6N) with over 30-fold expansion and high viability (Figure 6O). The elegance of this simple system enabled to expand and culture the TICs up to 10 passages with neither viability issues (>95% live), nor phenotypic changes. Upon growth factor removal, the TICs successfully differentiated into neuron and glial cells, expressing Tuj1+ and GFAP+, respectively (Figure 6P). This study highlights how the combination of a simple extrusion system and the appropriate choice of biomaterials were able to relevantly support the expansion, phenotype, and differentiation of primary-derived tumor stem cells (Li et al., 2018b).

While still in its infancy, 3D bioprinting warrants significant advances in the field by enabling both heterogeneity and complexity. With the ability to print multiscale ECM-like biomaterials, heterogeneous and more comprehensive tumor microenvironments that include gradients can be reproducibly recreated (Albritton and Miller, 2017). Ideally, bioinks that present a high degree of physicochemical functionalization may be preferred for the printing of patient-derived tissues, so that stiffness and additional tumor ECM may be tailored to more closely mimic native microenvironments.

### System-Based Approaches

One of the challenges in the culture of 3D culture models resides in static systems. In fact, while 3D tumors initially resemble *in vivo* samples, the lack of a dynamic microenvironment rapidly impacts cell proliferation due to mass transport limitations (Hirt et al., 2015). Dynamic systems such as rotary cell culture system (RCCS) bioreactors have been widely used in the general field of tissue engineering (Martin et al., 2004) and offer improved mass transfer and shear stress. In tumor engineering, such parameters are critical to recapitulate the native microenvironment that experiences local mechanical stresses. Similarly, this can be achieved by microfluidic systems (Sung and Beebe, 2014). With perfusion, the system provides continuous nutrient supply and waste removal, in turn maintaining a more stable culture environment, and enables to quantify transport parameters more readily (Avendano et al., 2019). With advanced microfluidic systems, such as organs-on-a-chip (Bhatia and Ingber, 2014), such properties can be combined with additional stromal components enabling the study of drug responses in dynamic contexts that incorporate spatiotemporal and biochemical heterogeneities (Table 3).

**Table 3:** Overview of microfluidic-based tumor models using patient-derived materials.

**Main cancer type**	**Purpose and application**	**Tumor model used**	**Patient numbers**	**Supporting matrix**	**Stromal cell components**	**Device**	**Maximum culture time**	**References**
Glioblastoma	Drug response	PDS	3	PEGDA	–	Custom built (glass)	14 days	Akay et al., 2018
Head and neck cancer	Radiation response	PDMEs	18	–	–	Custom built (PEEK)	68 h	Kennedy et al., 2019
Head and neck cancer	Radiation response	PDMEs	5 (3 primary, 2 metastasis)	–	–	Custom built (PDMS)	48 h	Cheah et al., 2017
Head and neck cancer	Radiation response	PDMEs	35	–	–	Custom built (glass)	72 h	Carr et al., 2014
Intestinal cancer	ICB profiling	PDMEs	1	Rat tail collagen-I	–	DAX-1, AIM BIOTECH	9 days	Aref et al., 2018
Liver cancer	Immunoresponse	HepG2 organoids (cell line)	–	Rat tail collagen-I	Monocytes and HBV-specific T cells	Custom built (PDMS)	24 h	Lee et al., 2018
Lung cancer	Immunoresponse	PDMEs	1	–	Tumor matched primary TILs	Custom built (COC)	4 days	Moore et al., 2018
Lung cancer	Biological studies, drug response	H1975 2D cells (cell line)	–	–	Primary airway and alveolar epithelial cells, primary lung microsvaculature endothelial cells	Custom built (PDMS)—wells coated with ECM (laminin, fibronectin, collagen-I)	28 days	Hassell et al., 2017
Lung cancer	Chemotherapy response	Single cell suspensions	8	Culturex BME	–	Custom built (PDMS)	48 h	Xu et al., 2013
Lung and squamous cancers	Chemotherapy response	Epithelial PDS	3	–	Primary pericytes	Custom built (PDMS)	3 days	Ruppen et al., 2015
Lung cancer	Chemotherapy response	PDMEs after xenografting	1	–	–	Custom built (PDMS)	10 days	Holton et al., 2017
Melanoma	ICB profiling	PDMEs	>20	Rat tail collagen-I	–	DAX-1, AIM BIOTECH	3 days	Jenkins et al., 2018
Mesothelioma	Chemotherapy response	PDOs (variable sizes)	2	HA/Gelatin	–	Custom built (PS, glass)	14 days	Mazzocchi et al., 2018
Multiple Myeloma	Chemotherapy response	MM single cells	7	Bovine collagen-I	Mesenchymal cells	μ-slide Chemotaxis 3D Ibitreat, IBIDI, LLC	7 days	Khin et al., 2014
Multiple Myeloma	Biological studies	MM single cells	3	–	Osteoblast cell line (hFOB 1.19)	Custom built (PDMS)	21 days	Zhang et al., 2014
Multiple Myeloma	Biological studies	MM single cells	9	–	Osteoblast cell line (hFOB 1.19)	Custom built (PDMS)	21 days	Zhang et al., 2015
Multiple Myeloma	Drug response	MM single cells	17	–	CD138- bone marrow stromal cells	Custom built (PDMS)	3 days	Pak et al., 2015
Ovarian cancer	Chemotherapy response	PDMEs after xenografting	2	–	–	Custom built (PDMS)	8 days	Astolfi et al., 2016
Pancreatic cancer	Immunoresponse	PDMEs	5–10	Rat tail collagen-I	–	DAX-1, AIM BIOTECH	24 h	Wang et al., 2018a

*BME, basement extract membrane; COC, cyclic olefin copolymer; ECM, extracellular matrix; HA, hyaluronan; ICB, immune checkpoint blockade; MM, multiple myeloma; PDME, patient-derived microdissected explants; PDMS, polydimethylsiloxane; PDOs, patient-derived organoids; PDS, patient-derived spheroid; PDX, patient-derived xenograft; PEGDA, poly-(ethylene glycol) diacrylate; PEEK, polyether ether ketone; PS, polystyrene*.

#### Bioreactors

RCCS bioreactors have been widely used primarily to facilitate the self-assembly and culture of scaffold-free spheroids (Ferreira et al., 2018). Yet, bioreactors have been most often used *post* production for patient-derived tumor models to prolong the life and predictive power of 3D tumor models, in the context of drug screening. Physical bioreactors include roller tuber, spinner flask, gyratory shakes, and microgravity bioreactors (Saglam-Metiner et al., 2019) or obtain shear via perfusion systems, with the general purpose of increasing fluid transfer by convection, ultimately improving mass transfer (Selden et al., 2018). In the context of patient-derived 3D models, both RCCS and perfusion systems were used in the field of multiple myeloma (Ferrarini et al., 2013; Belloni et al., 2018), breast cancer (Muraro et al., 2017), colorectal cancer (Manfredonia et al., 2019), and glioblastoma (Li et al., 2018b), overall enhancing the viability of the culture systems.

**Figure 7:**
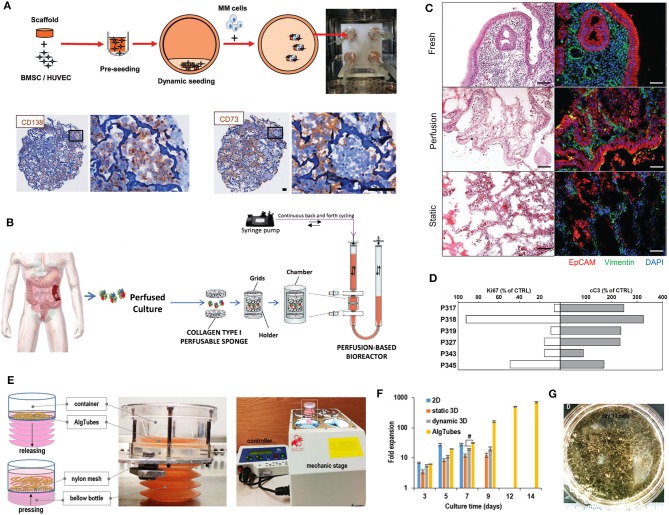
Bioreactor systems with patient-derived materials and/or primary cells. **(A)** Generation of a 3D multiple myeloma (MM) microenvironment in a rotary cell culture system (RCCS^TM^) bioreactor; scaffold is pre-seeded *in vitro* with bone marrow stromal cells (BM-MSCs)/endothelial cells (HUVECs) and transferred to the RCCS bioreactor. MM cells are then added and cultured dynamically up to 7 days. IHC shows uniform distribution of CD138+ MM cells and CD73+ stroma. Scale = 100 μm. **(B)** Schematic representation of the experimental design. For perfused cultures, colorectal cancer specimens (*n* = 3/bioreactor) were placed between two collagen type I discs within a ring-shaped holder, restrained by two grids on the top and bottom. The holder was then inserted in the bioreactor chamber and subjected to continuous alternate perfusion. **(C)** Immunostaining shows architectures and protein expression in perfused tissues similar to fresh tissues [EpCAM (red), vimentin (green), DAPI (blue)]. **(D)** Percentages of proliferative (Ki67+) and apoptotic (cC3+) cells in perfused cultures supplemented with 5-FU relative to untreated tissues for tissues from 6 individual patients, showing high heterogeneity in response. **(E–G)** A prototype bioreactor for scalable glioblastoma tumor-initiating cells (TIC) production in alginate tubes (AlgTubes). **(E)** The bioreactor contains a cylindrical container and a plastic bellow bottle, which are separated by a nylon mesh. AlgTubes with TICs are suspended in the cylindrical container and the medium is stored in the plastic bellow bottle that can be pressed to flow the medium into, or released to withdraw the medium from the container. The mechanic stage is used to press and release the bellow bottle. The controller can be programmed for the pressing and releasing speed, as well as the duration of the interval between the pressing and releasing. **(F)** Fold-expansion of glioblastoma TICs cultured in AlgTubes, 2D, static 3D, and dynamic 3D suspension. **(G)** Image of glioblastoma TICs harvested from the bioreactor on day 10. **(A–G)** reproduced with permission from Belloni et al. (2018), Manfredonia et al. (2019) and Li et al. (2018b), respectively. ^#^*P* < 0.05.

Specifically, in a study by Ferrarini et al. (2013), a RCCS^TM^ bioreactor was used to culture various multiple myeloma PDMEs (2–3 mm^3^ in size) from various metastatic sites. Histological examination demonstrated conservation of viable myeloma cells within their native microenvironment, with a well-conserved histological architecture that included bone lamellae (when relevant) and vessels. The use of dynamic culture for 7 days was particularly important to the maintenance of the vessels, which overall architecture was otherwise disrupted and disappeared in static culture. A further 3-day treatment with bortezomib, a standard anti-myeloma drug, showed that the drug-treated samples displayed an overall concordance in the response to the drug *ex vivo* and *in vivo*. Notably, *in vivo* drug resistance seen for one of the patients treated was also observed for the corresponding explant in the bioreactor system (Ferrarini et al., 2013). In a follow-up study by the same group (Belloni et al., 2018), the authors focused on isolated MM cells. But here, the authors used a scaffold-based approach, Spongostan sheets, a sponge derived from porcine gelatin (Ethicon, Inc.), pre-loaded with patient-matched BM-MSCs and HUVECs. The co-culture promoted the survival of isolated primary MM cells for up to 7 days in the bioreactor (Figure 7A). The pool of allogeneic BM-MSCs, HUVECs and MM cells retrieved from the scaffolds at the end of culture matched the input number, indicating that the cells survived but did not proliferate. IHC showed uniform distribution of MM cells and CD73+ stroma. For 6 patients used in culture in the bioreactor, both MM cells and stroma retained their specialized functions and relevant chemotherapeutic responses. Overall, the *ex vivo* 3D co-culture model in bioreactor met the requirements of recapitulated MM-BM dialogue, permanence, and survival of primary MM cells for an extended time period, thereby also incorporating a temporal dimension rarely seen in 2D and static systems of MM. This achievement allowed the dissection of clonal dynamics during MM progression and in response to therapy, a central issue in MM investigations. The combination of allogeneic BM-MSCs to match the patient multiple myeloma cells was another strength of this study, as it allowed for the recapitulation of the patient's bone marrow niche specificity (Belloni et al., 2018).

In breast cancer, Muraro et al. used a custom perfusion bioreactor to maintain breast cancer explants for up to 14 days (27 patients) (Muraro et al., 2017). Upon manual fragmentation of tumor specimens into 2 × 2 × 2 mm pieces, two 8 mm-scaffold discs made of collagen type I were used in a sandwich culture system to induce homogeneous tissue perfusion by the medium. The authors used next generation sequencing to validate a close match between clinical samples and the bioreactor-cultured explants. As a comparison, the tumor fragments from static cultures displayed significantly lower percentages of viable tumor cells. The maintenance of explants for up to 2 weeks enabled the assessment of anti-estrogen treatments and other antibody treatments. Subsequently, the same perfusion bioreactor concept (Figure 7B) was used to culture tumor fragments from colorectal cancer specimens, a cancer known to be more difficult to culture *in vitro* (Manfredonia et al., 2019). Contrary to the breast cancer study, the colorectal samples were cultured for only 3 days with the bioreactor. Compared to static cultures, the bioreactor-cultured specimen preserved tissue mass, higher tissue cellularity, and overall initial architecture, whereas it was lost in non-perfused cultures (Figure 7C). For instance, the epithelial component and immune cell subsets in perfused cultures were similar to fresh tissue but reduced in static tissues. Critically, highly heterogeneous responses were observed between patients (Figure 7D). Overall, these studies strongly demonstrated how bioreactor systems combined with scaffold systems have clear benefits for the maintenance and longer culture of primary tissue samples with the capacity to address heterogeneity (Cassidy et al., 2015; Dagogo-Jack and Shaw, 2017; Bocci et al., 2019). An important consideration is however the likelihood of different biomaterials surviving in a bioreactor microenvironment, where softer materials such as hydrogels may be torn a part in rotary well-culture systems. In this context, perfusion on a static system may be recommended.

In glioblastoma, a prototype bioreactor was developed for the scalable manufacturing of patient-derived glioblastoma cells, after extrusion in alginate hydrogel tubes (Figure 7E) (Li et al., 2018b). A mechanical stage enabled to compress the compartment containing the media, resulting in cyclic flow in the compartment containing the tubes. Compared to low expansion in static 2D/3D and dynamic free suspension in 3D, the cells within the alginate tubes were able to be expanded up to 14 days with a 710-fold expansion (Figures 7F,G) and high volumetric yield when placed in the bioreactor. This study represents a key advance in the rapid, cost-effective and scalable expansion of patient-derived cells, with significant impact for personalized high throughput drug screening, which require high cell numbers (Li et al., 2018b).

#### Microfluidics

Microfluidic 3D cell culture represents an optimum strategy to deliver more complex cancer microenvironments and investigate cancer dynamics. The concept of microfluidics allows researchers to culture and study cellular processes and drug responses in a miniaturized, yet well-defined and more biologically relevant culture environment (Holton et al., 2017). Suitable to study an array of cancer hallmarks, such as cancer proliferation, angiogenesis, migration, invasion, microfluidic devices enable multiple spatiotemporal layers of complexity. Numerous applications have used microfluidics to measure the response of tumor cells to quantifiable concentration of chemokine gradients (Xu et al., 2014). Both tumor and stromal cells indeed exhibit directional migration toward a chemokine source during growth and dissemination, which can be achieved by microfluidic platforms. Compared to the highly complex models used with cancer cell lines, patient-specific microfluidic models are relatively more modest, mainly using scaffold-free PDME/PDS/PDO approaches or simple co-culture models, used mostly for cytokine profiling or treatment assessment, and up to 28 days culture, and are presented hereafter.

##### Immunotherapy

**Figure 8:**
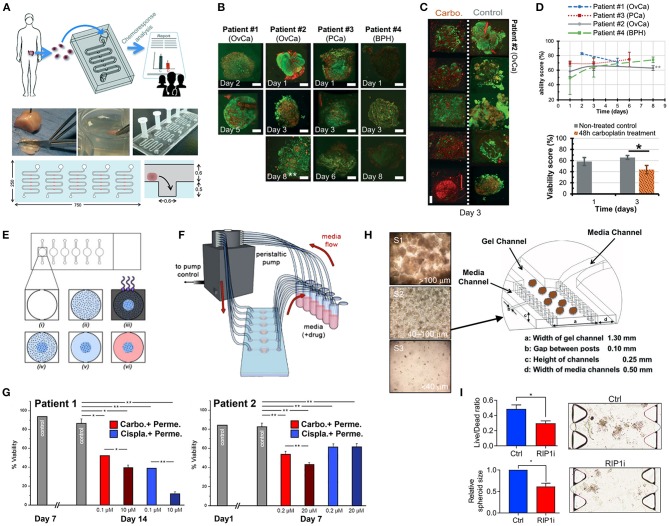
Microfluidic systems using patient-derived materials for chemotherapy **(A–G)** and immunotherapy **(H,I)**. **(A)** Top: schematic of a patient-derived tumor explant, microdissected, loaded in a microfluidic device and injected with various drugs. Results are then interpreted to identify non-responders to treatment for a personalized treatment strategy. Bottom: photograph of tumor prior and after microdissection in 500 μm-PDMEs and design of the microfluidic device containing up to 5 chambers for PDME entrapment and drug testing. **(B)** PDMEs from various patients and different cancers are analyzed by live/dead cell assay using confocal microscopy (green = live, red = dead), showing various PDME architectures and viability patterns. **(C)** PDMEs from an ovarian tumor treated with carboplatin shows high heterogeneity in PDME structure and viability response for PDME originating from the same tumor. **(D)** Corresponding graphs of viability data from **(B)** (top) and **(C)** (bottom) compared to non-treated controls show inter-cancer tumor heterogeneity and drug efficacy on PDMEs from one tumor, despite intra-tumor heterogeneity observed in **(C)**. **(E)** Microfluidic device enabling *in situ* spheroid patterning technique: a microfluidic chamber (i) is filled with a mixture (blue) containing hydrogel precursors, photoinitiator, and patient-derived tumor cells (ii) and then illuminated with UV light through a photomask (gray) (iii). Exposed precursor is crosslinked into a hydrogel (dark blue), detaining cells within the region (iv), and non-crosslinked gel is flushed form the chamber with clean saline from the chamber (v). Finally, saline is replaced with media (red) (vi) for incubation. **(F)** Schematic of system that offers low volumes and closed loop fluidic circuit controlled by a computer-controlled peristatic pump to treated individual organoids. **(G)** *In vitro* chemotherapy assessment of organoids derived from two patients with mesothelioma treated with various drug doses and combinations shows different response between patients, although treatment occurred at different times between patients (viability measured from live/dead confocal microscopy on organoids). **(H)** Schematic of PDMEs obtained after dissection and sieving. Fraction 2 (40–100 μm) is usually used in microfluidic devices such as the 3D cell culture chip from AIM Biotech shown, which contains a center gel region. Gel loading port and media ports labeled **(B)**, along with center and side channels **(C)**. **(I)** The AIM Biotech microfluidic device was used by Wang et al. to test a selective RIP1 inhibitor on pancreatic ductal adenocarcinoma PDMEs. After RIP1i treatment (*n* = 5 patients shown), PDME live/dead ratio, and size decreased. **(A–I)** reproduced with permission from Astolfi et al. (2016), Aref et al. (2018), Mazzocchi et al. (2018), and Wang et al. (2018a), respectively. **(D) Top:** **Result for PDMEs stained and imaged a second time; **Bottom:** *p-value = 0.014. **(G)** *p < 0.05, **p < 0.01. **(I)** *p < 0.05.

One specific patient-derived application using microfluidic devices is the modeling of the dynamic response to ICB in immuno-oncology. Immune checkpoint pathways can indeed be co-opted by cancer to evade immune response and drugs, while interrupting immune checkpoints can be an effective way to boost anti-tumor immunity and prompt cancer regression (Topalian et al., 2015). Microfluidic devices are an elegant option to assess the response of PDMEs against various ICB-related inhibitors, due to the presence of native immune cells. In a study by Aref et al. for example, using enzymatic digestion, tumor specimens from various tumors were dissociated into single cells, PDS, and macroscopic PDMEs (>100 μm). The spheroid fraction was mixed with collagen-I and used for culture in a cyclic olefin co-polymer (COC)-based 3D microfluidic device (DAX-1, AIM BIOTECH) (Aref et al., 2018). In that study, a small intestinal neuroendocrine tumor was cultured up to 9 days for RNA-sequencing and cytokine profiling. This study was a follow-up by the same group, who used a similar microfluidic approach to culture melanoma PDMEs from a higher number of patients (>20) (Jenkins et al., 2018). Lymphoid and myeloid cell populations were maintained in organoids from various cancer types and the PDS adequately responded to ICB. One of the limitations of these systems resides in the inability to recapitulate T-cell priming or recruitment of naïve immune cells to the tumor microenvironment. This could be addressed in the future by designing more complex tumor-on-a-chip platforms that provide a source of immune cells for interactions with the PDMEs. Additionally, traditional microfluidic devices usually employ polydimethylsiloxane (PDMS) which adsorbs small hydrophobic molecules, likely influencing drug testing. In this context, the use of COC-derived microfluidic devices may be more suitable. The COC-derived microfluidic device was also recently used for PDME from pancreatic ductal adenocarcinoma (PDA) (Wang et al., 2018a) prepared similar to Jenkins et al. (2018) and Aref et al. (2018) (Figure 8H) ICB studies. After mincing of the explant and resuspending the PDMEs in collagen, the mixture was inserted into the DAX-1 microfluidic device before assessing a novel inhibitor molecule (RIP1i) (Figure 8I). The use of the microfluidic device for the PDA-derived PDMEs enabled the assessment of reproducible treatment with RIP1i, and the profiling of a spectrum of immunogenic cytokines of up to 10 patients, which corroborated the results from animal model experiments.

##### Chemotherapy

Microfluidic chambers often allow a high number of replicates that are tested on several fragments of specimens from the same tumor piece, with the overall possibility to extend explant viability due to perfusion. In a study by Astolfi et al. (Figures 8A–D), large PDMEs extracted from ovarian (2 patients) and prostate (1 patient) cancer were successfully cultured inside a PDMS microfluidic device for up to 8 days, with no decreased of viability over time (Astolfi et al., 2016). Four PDME types displayed heterogeneous staining patterns with the non-cancerous tissue being the least viable of all 4, possibly due to reduced metabolism (Figures 8B,D). Subsequently, one of the ovarian PDMEs was treated with carboplatin after 24 and 48 h inside the device, at a dose equivalent to the maximum theoretical blood concentration of the drug in a normal patient treated. Due to tissue availability and the high number of microfluidic chambers, a total of 25 PDME replicates were loaded in the device to ensure that intratumoral heterogeneity could be addressed. Specifically, high variability between PDMEs was observed (Figures 8C,D), which the authors attributed to a variable chemoresponse of different cell subpopulations within the tumor tissue, as ovarian tumors are known to exhibit high intratumoral heterogeneity. Ultimately, despite high variance, the patient response to the treatment corroborated the *in vitro* results. In some cancers, the use of PDMEs is impractical due to tumors that are either not stiff enough or too dependent on the microenvironment for survival. In this case, xenografting may be used to expand the tumor mass prior to excision, fragmentation, and culture in a microfluidic device. Holton et al. used this strategy for lung, bladder, and melanoma explants (Holton et al., 2017). After mouse excision, the PDXs were dissociated by fine needle aspiration and cultured in a continuous perfusion microfluidic device for up to 10 days. In this study, the lung-derived patient PDXs were dissociated in 18 PDME samples and used for treatment with staurosporine (a broad protein kinase inhibitor) for 5 days. The PDMEs showed significantly reduced viability compared to non-treated controls, and displayed only slight intratumoral heterogeneity, providing more chance of success when clinically translated (Holton et al., 2017).

It is known that cancer cell lines respond differently than primary tumor cells to chemotherapeutic agents. This was evidenced in lung cancer using a microfluidic chip-based 3D co-culture device (Xu et al., 2013). After isolation of primary tumor cells from fresh lung tumor specimens, the cells were co-cultured for 24 h with cell-basement membrane extract and submitted to drug testing. When cancer cell lines were used instead of the primary cells for co-culture, the IC50 of gefitinib was much larger for primary cancer cells. Overall the apoptosis rates were similar between the 8 patients tested. However, it must be noted that this study was looking at individual cells, with some cell aggregates (Xu et al., 2013). In a study by Mazzocchi et al. (2018) (Figures 8E–G), tumor cells were derived from mesothelioma (2 patients), which were grown *in situ* into organoids of high cellular viability in HA-gelatin hydrogels. Organoids were observed after 1 and 7 days for each patient, upon which two different doses of chemotherapeutic mixtures carboplatin/pemetrexed or cisplatin/pemetrexed were injected. Different responses were observed for each patient after a further 7 and 14 days according to the cocktails of drugs and doses selected, highlighting the intrinsic patient differences in response to similar treatments. A non-traditional microfluidic device was also recently presented by Akay et al. (2018), where various drug concentrations were able to be tested simultaneously (7 channels containing up to 11 microwells) on glioblastoma PDS and effectiveness was measured by spheroid size and viability. From the three patients tested, large interpatient heterogeneity was observed, although the same decreasing trend was observed for 4 out of the 7 channels tested. This method offers high-throughput testing, as it allows researchers to simultaneously treat organoids with various drug concentrations.

In a study by Ruppen et al. PDS were formed using primary lung adenocarcinoma cells from two patients, using a cell gravity microwell-entrapment system (Ruppen et al., 2015). After the first 24 h of spheroid formation, their size decreased due to compaction. In a variant, the epithelial cells were injected with primary pericytes at a 5:1 ratio, as to assess the known drug barrier effect from pericytes. Spheroids again formed homogeneously and when treated with various cisplatin concentrations, the tumor/pericytes spheroids were significantly less chemosensitive, validating the known effect of pericytes using this microfluidic device.

##### Radiotherapy

In some cancers such as HNSCC, the standard treatment strategy involves gamma irradiation. As a result, microfluidic devices have been developed not only to maintain the viability of tumor specimens but also to sustain irradiation. The microfluidic devices used for the irradiation of HNSCC PDME samples have consisted of mostly PDMS (Cheah et al., 2017), but also glass (Carr et al., 2014) or more recently, polyether ether ketone (PEEK) (Kennedy et al., 2019). In the study with the largest number of patients (Carr et al., 2014), the specimens of 35 patients were sectioned into 3 mm^3^ PDME samples and loaded in the microfluidic device for 72 h of culture. The study showed increased apoptotic index with increasing Gy dose, but when clinical doses were used, cell death decreased after 22 h. In a subsequent study (Cheah et al., 2017), the PDMEs from 5 HNSCC patients (3 primary and 2 metastatic) were tested from 0 to 20 Gy but only left in culture for 24 h following irradiation. Interestingly, whereas metastatic samples were expected to be more resistant to irradiation, two out of three of the metastatic PDMEs had higher responses following a 15 Gy dose compared to non-metastatic samples. Overall, the PDMEs from the 5 patients displayed very variable responses to irradiation from none to mild, confirming intertumoral, and intratumoral heterogeneity. These results emphasize the value of individual analysis of tumors, combined with a high number of technical replicates per patient, to truly determine patient specific response (Cheah et al., 2017). In a recent study by Kennedy et al. a PEEK-derived microfluidic device was used to load freshly excised samples from 18 patients (Kennedy et al., 2019). The specimens were cut using vibratome slicing and cultured for 68 h with 2 h interval perfusions. The specimens were further submitted to 2 Gy irradiation ± Cisplatin, which denotated increased apoptotic staining compared to the controls. Intratumoral heterogeneity was evident in all of the immunohistochemistry markers before and after irradiation treatments. While the advantage of this PEEK system resides in an easy-to-use setup with the possibility to assess irradiation-related effects, the microfluidic device comprised only 4 chambers, limiting the number of replicates being investigated simultaneously (Kennedy et al., 2019).

#### Tumor-on-a-Chip

In the last decade, numerous tumor-on-a-chip systems, deriving from organ-on-a-chip systems, have shown great potential in providing the complexity of various dynamic aspects of the cancer while also incorporating high-throughput techniques (Caballero et al., 2017). These systems rely on microfluidics approaches and combine the advantages of individual tumor models, by offering multicellular architecture, tissue-tissue interface, and a biomimetic physical microenvironment that can sustain vascular perfusion (Sontheimer-Phelps et al., 2019). Yet, dynamic cancer processes such as invasion, migration, intravasation, extravasation, and metastasis models have been developed mostly using cell lines. Only recently have studies combined patient-derived tumor materials and stroma toward the development of more complex “personalized tumor-on-a-chip” systems, yet often combining cell lines in the process. Compared to the previous section which reported simple microfluidic systems used mostly to assess cytokine profiling/therapy on single-cell-type-derived spheroids or explants, the following section reports more complex systems (mimicking the immune system and metastasis) that combine various cell types for therapy response as well as biological studies.

##### Immune-system-on-a-chip

**Figure 9:**
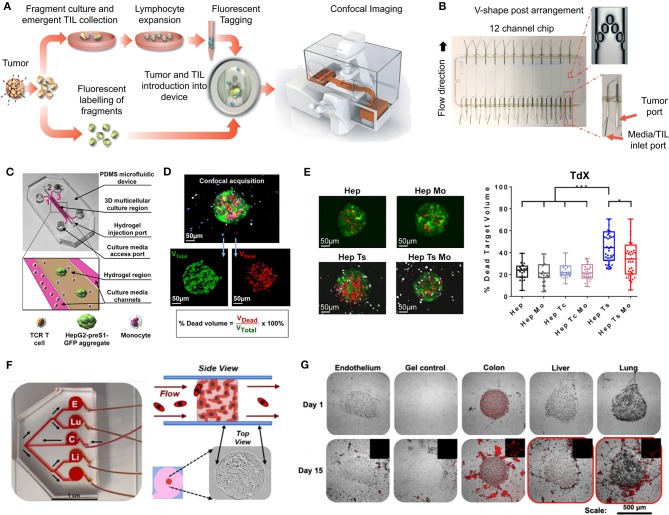
Tumor-on-chip systems. **(A)** Diagram illustrating the processing steps involved in the preparation of patient-derived tumor cells and tumor-infiltrating lymphocytes (TILs) from the same tumor sample, and live imaging by confocal microscope. **(B)** 12-channel multiplexed cyclic-olefin-copolymer (COC)-based microfluidic device with V-trap design for capturing tumor sample in flow stream and dual port entry for TILs. **(C)** A 3D multicellular tumor microenvironment microfluidic model consisting of a middle hydrogel channel (2) surrounded by two media channels (1, 3) for the mechanistic study of the effect of monocytes on T cell receptor-redirected T cell (TCR T cell) killing of tumor cell aggregates. Human monocytes were inserted together with target HepG2-preS1-GFP cell organoids in collagen gel in the central hydrogel region (2), while hepatitis B virus (HBV)-specific TCR T cells were added into one fluidic channel (1) to mimic the intrahepatic carcinoma environment. **(D)** Representative confocal image of a target cell organoid (in green) surrounded by monocytes (in blue) and HBV-specific TCR T cells (in white), in which the presence of dead target cells is DRAQ7+. **(E)** Left: Representative target cell HepG2 cell organoids (Hep) cultured with monocytes (Mo) and/or HBV-specific T cell (Ts), in which the presence of dead target cells is DRAQ7+(in red). HBV-specific TCR T cells are labeled with Cell tracker violet dye (in white), while monocytes are unlabeled. Right: Box plot of the percentage of dead target volume after 24 h of co-culture with retrovirally transduced (Tdx) HBV-specific TCR T cells (Tc = control T cell). **(F)** Metastasis-on-a-chip device and *in situ* tumor and tissue construct biofabrication. Arrows show fluid flow (E, endothelium; Lu, Lung; C, colorectal cancer organoids made of RFP tagged HCT116 cells; Li, liver; blank, control). Constructs comprised of cells in ECM hydrogels exist under fluid flow and have the capability to experience circulating cells either interact or pass by. **(G)** Metastasis tracking at day 1 and day 15 showing HCT116 cells colonizing other organs, using phase and epifluorescence microscopy **(A–G)** reproduced with permission from Lee et al. (2018), Moore et al. (2018), and Aleman and Skardal (2019), respectively. *P ≤ 0.05, ***P ≤ 0.001.

It is generally acknowledged that in the arena of cancer modeling, the immune response has been relatively neglected, due to the complexities of recapitulating it *in vitro* (Polini et al., 2019). Yet, tumors-on-a-chip provide a relevant technology that can pave the way toward this direction, as they can possibly offer a mean to overly study inflammation (Han et al., 2012) and immune cells-tumor interactions by combining patient-derived materials with cell lines. For instance, Moore et al. developed a COC-derived microfluidic model termed EVIDENT (*ex vivo* immuno-oncology dynamic environment for tumor biopsies) enabling the accommodation of 12 separate biopsy fragments for interaction with patient-matched flowing tumor-infiltrating lymphocytes (TILs) (Figures 9A,B) (Moore et al., 2018). The EVIDENT microfluidic system displayed quantifiable levels of TIL infiltration and tumor death, mimicking *in vivo* tumor response to ICB treatment of flowing TILs. Innovatively, the system used a material with high optical transparency and was loadable onto the stage of high resolution confocal microscope enabling real-time image acquisition and analysis (Moore et al., 2018). While the method was established with cell lines, the study also assessed one NSCLC patient sample. At 24 h post TIL administration, the treated NSCLC tumor fragment displayed substantial TIL infiltration with proximal cellular apoptosis and was time-dependent. Other studies have focused on recreating cell line tumor organoids and used a microfluidic device to test the response upon addition of differentiated patient-derived immune cells. For example, Lee et al. developed an intrahepatic tumor microenvironment model to investigate the immunosuppressive potential of monocytes toward Hepatite B virus-specific T cells (differentiated from peripheral blood mononuclear cells) and the role of ICB signaling using a static 3D microfluidic model (Figures 9C,D) (Lee et al., 2018). The benefit of using the microfluidic device, beyond the 3D micro-chamber, was to allow sequential injection, with first HepG2 cell lines aggregates and patient-derived monocytes, then followed by the patient-derived T cells. It was shown that functional differences existed among differently produced T cells, where monocytes suppressed only retrovirally transduced T cell cytotoxicity toward cancer cells while cytotoxicity was not affected by the presence of monocytes. This result was only observed in the microfluidic device (dynamic 3D) (Figure 9E) and not in a static 2D setting.

##### Metastasis-on-a-chip

The dynamic process of metastasis is highly suitable for study using microfluidic platforms (Caballero et al., 2017), and was largely investigated for bone metastasis. This was investigated heavily for MM since primary MM cells are easily accessible and could be injected easily in microfluidic devices comprising bone-like tissues. Zhang et al. exploited this strategy by creating what the authors referred to as a “3D ossified” tissue (Zhang et al., 2014). This study is highly cited in the field of microfluidics, as reported in many reviews (Bhatia and Ingber, 2014; Carvalho et al., 2015; Fong et al., 2016a; Arrigoni et al., 2017; Peela et al., 2017; Rothbauer et al., 2019; Sakthivel et al., 2019; Sontheimer-Phelps et al., 2019), yet the limitations of the study escaped most of them. The “3D ossified” “tissue” appellation was merely disproportionate; the “tissue” consisted simply of a monolayer of human osteoblasts (hFOB 1.19 cell line) cultured on the flat surface of microfluidic chambers for 4 days prior to the pumping of bone marrow mononuclear cells from MM patients for 4 h, followed by perfused culture for 21 days. Due to the improper “scaffold” terminology used in the paper, it was often wrongly assumed that a physical 3D scaffold was employed to grow the tissue. Structurally, the osteoblast-derived tissue was <60 μm (i.e., actually 2D). Additionally, no characterization of bone ECM markers, critical to bone metastasis, was performed, although CD138+ and CD38+ CD56+ populations were capable of proliferating for 7 days on top of the osteoblast layer before stopping proliferation and forming colonies in the 7–21 days range. The authors stated that less mineralization took place in the presence of MM cells, but this arose from basic visual inspection, without quantitative measure (Zhang et al., 2014). A follow-up study by the same group sought to provide more mechanistic discussion regarding the osteoblasts/MM cells interactions and showed how N-cadherin from osteoblasts contributed to the homing and retention of MM cells onto the osteoblast layer. In this study, the authors described how to maximize long-term maintenance of co-cultured primary MM (Zhang et al., 2015). Further work interrogated bone stroma/MM cells interactions and drug responses in microfluidic devices (Khin et al., 2014; Pak et al., 2015), using solely primary cells, yet the bone marrow microenvironment reflected simple 2D co-culture models aided by microfluidic technologies, rather than “tumors-on-a-chip” systems. Interestingly, in one study investigating 17 patients (Pak et al., 2015), response to the proteasome inhibitor bortezomib was clinically matched with the response from the *in vitro* model, but only when the MM cells were co-cultured with CD138- bone marrow stromal cells present. This achievement questions the necessity of having complex structural 3D microenvironments.

Recently, ingenious multi-site metastasis-on-a-chips, deriving from multi-organ-on-a-chip technologies (Skardal et al., 2017), have combined photopatterning of HA-gelatin hydrogels and microfluidics, to recreate various types of organoids in individual chambers, including endothelium, lung, and liver (Figure 9F) (Aleman and Skardal, 2019). By culturing upstream organoids of colorectal cancer under recirculating fluid flow for up to 15 days, fluorescently-tagged tumor cells were tracked when they detached from the colorectal cancer organoids and metastasized in the organs from other chambers, homing preferentially to liver and lung (Figure 9G), as seen clinically. While this study was entirely performed with cell lines (HCT116), the use of similar systems with patient-derived primary tumor cells hold potential for a more holistic approach to assess individual metastatic prevalence and personalized therapy selection.

## Perspective

**Figure 10:**
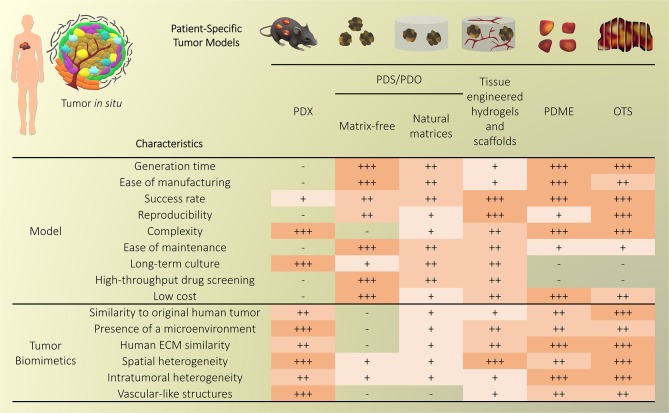
Advantages and disadvantages of current patient-specific tumor models.

Advances in 3D cell culture have led to novel discoveries, including specific details that occur during cancer development and progression that had previously remained unknown. Each 3D model comes with its own advantages and limitations (Figure 10), although typically, no model can answer all questions, thereby, a multi-model approach seems most sensible to study cancer heterogeneity. The inability to provide representative preclinical platforms that are patient-specific is, today, one of the key frontiers impairing personalized and effective cancer treatment. Encouragingly, 3D tumor modeling has made significant progress in this direction by combining advanced modeling technologies with innovative biomaterials that can partly mimic the heterogeneous context of real tumors (Fong et al., 2016a; Peela et al., 2017). Unfortunately, these systems have reduced translational power by failing to systematically use patient-derived materials as these present with limited tissue access, tissue quantity and viability *ex vivo*. In the arena of *ex vivo* culture of patient-derived tumors, efforts are still today largely focused on the use of scaffold-free/Matrigel approaches using predominantly PDOs and PDS (Nagle et al., 2018), that allow minimal processing/engineering and rapid drug assessment. While this strategy offers advantages in terms of simplicity, yield, minimal labor, and are relatively high-throughput, all of which appealing to pharmaceutical companies, these models suffer from batch-to-batch heterogeneity and moreover lack the supportive 3D stromal network that is critical in enabling heterogeneity considerations.

First, it is necessary to delineate the purpose of each system, and the key components related to the research questions. For example, vascularized networks in the assessment of metastasis, increased viability in the case of long-term drug testing or multiple stroma components in the assessment of drug resistance. The determinants of tumor growth highly depend on tumor type, hence it is imperative to use specific tools that mimic individual contexts (Thoma et al., 2014), in terms of cellular and non-cellular components, as well as physical/chemical cues. Innovative biomaterials and tissue engineering strategies, coupled with manufacturing technologies such as bioprinting, currently enable researchers to adequately mimic disease-specific contexts by providing stiffness, architectures, and chemical compositions specific to local organs. Yet, the full potential of these techniques has not been sufficiently exploited in the context of patient-derived components, requiring the adoption of a new mindset that allows heterogeneity to be an intrinsic part of tumor models. Biomaterial-based models allow for the effective support of heterogeneous cultures, bringing forth a degree of complexity in 3D cultures that was previously unachievable. However, due to a lack of inherent factors, scaffold design is key to sustaining patient-derived samples.

Synthetic and semi-synthetic hydrogels for instance have the power to be tuned to replicate local ECM libraries, with the possibility to incorporate growth factors, MMPs, and RGD motifs specific to each microenvironment, and hold promise to mimic a high spectrum of heterogeneities. For instance in our own work, we have shown how PEG-Heparin hydrogels represent a modular platform for systematic angiogenesis modeling by incorporating a variety of growth factors, ligands, and cleavable peptide linkers for prostate and breast cancer modeling (Bray et al., 2015). Similarly, semi-synthetic gelatin-methacryloyl (GelMA)-derived hydrogels are attractive photocrosslinkable hydrogels that offer a high degree of physicochemical functionalization and properties (Yue et al., 2015; Loessner et al., 2016; Meinert et al., 2018). In the Hutmacher group, Kaemmerer et al. have indeed shown how they could be a suitable platform for the growth of ovarian cancer spheroids, investigating the effects of key tumor ECM components (laminin and hyaluronan) and matrix stiffness (ranging from 0.5 to 9 kPa) which both revealed significant responses in growth and treatments of organoids (Kaemmerer et al., 2014). Subsequently, GelMA gels were successfully used to model breast cancer invasion and chemoresponse *in vitro* with cell lines (Donaldson et al., 2018). Recently GelMA gels were also able to modulate the production of pro-inflammatory cytokines, TNF-α, by human mononuclear cells (Donaldson et al., 2018). Such property is of high interest when addressing immunoresponse *in vitro*. The ease of use with GelMA gels is also attractive to bioprinting, as specific patterns with different compartments, could be bioprinted followed by simple UV or light crosslinking (Pepelanova et al., 2018). Ultimately, using such hybrid biomaterial systems provides a versatile tissue culture platform that addresses the limited bioactivity of synthetic matrices while controlling batch-to-batch physical properties that critically influence each tumor microenvironment. Combining the physicochemical versatility of GelMA with the tailorability of bioprinting (multiple components printed simultaneously; Ke and Murphy, 2019; Meng et al., 2019), will offer tremendous opportunities to recreate complex biomaterial composite platforms that account for heterogeneous tissue level organization without losing control over relevant biochemical and biophysical cues, as seen in explants. 3D bioprinting is further advantageous as it can reconstruct complex structures from digital designs that can be patient-specific and has upscaling potential (Ma et al., 2018). Yet, it must be noted that the viability of patient-derived materials may be compromised by the printing process and so manufacturing systems that are rapid, mild, and cell-friendly should be chosen in this context. The incorporation of decellularized tumor matrices may be another option that enables heterogeneity recapitulation. To date, many studies have shown how such matrices led to very useful 3D tumor models for breast (Jin et al., 2019; Liu et al., 2019), skin (Brancato et al., 2018), and colon cancer (Hoshiba and Tanaka, 2016; Pinto et al., 2017; Romero-López et al., 2017), yet they are still to be used in co-culture with patient-derived tumor cells.

So where do we go from here? In order to mimic an organ or tissue, a combination of expertise from chemists, biologists, and biomedical engineers will be required to manufacture a more *in vivo*-like tumor microenvironment to give context to the spheroids, and/or to support the culture of multiple supporting cell types derived from the original organoids (Foley, 2017). In this context, the use of CAFs is one important component which can be relatively easily incorporated to models, but efficiently raise the heterogeneity profile of tumor models (Augsten, 2014). More problematically, tissue-specific endothelial cells and vessel-supporting cells are critical to establish patient and organ-specific vascularization, which has considerable downstream effects on tumor cell survival and metastasis, with implications on access to nutrients and therapeutics. Such primary cells are however difficult to collect, expand and maintain in a 3D setting and the community has traded this aspect of heterogeneity for well-characterized HUVECs.

Finally, biotechnologies, such as bioreactors (Selden et al., 2018), microfluidics (Shang et al., 2019), and tumor-on-chip (Rothbauer et al., 2019; Sun et al., 2019) approaches represent exciting options to raise heterogeneity by offering integrative platforms for controlled dynamic co-cultures, including relevant physical and chemical gradients specific to individual microenvironments. The use of patient-derived components, either tumor or stromal derived, combined with supportive scaffold biomaterials integrated into such dynamic platforms may offer the highest degree of heterogeneity, and hence relevance, in tumor models (Esch et al., 2015). In the specific context of metastasis, the multi-site organs-on-chips are relevant candidates for increased complexity (Skardal et al., 2016a,b, 2017; Aleman and Skardal, 2019), although it may be impossible to recreate fully patient-specific micro-organs with this strategy. In this case, a mixture of organ-specific cell-lines derived organoids with a primary patient-derived tumor still hold great potential for metastasis assessment and personalized chemotherapeutic guidance. In addition, in the event of multi-cellular or multi-organ model development, a question as to how to enlarge the models remains. Due to nutrient and oxygen deprivation as researchers develop real-sized tumors and their matrix *in vitro*, cell/tissue death and necrosis will inevitably occur. The need for the implementation of blood vessels and other structures are required to be a part of the tumor model, leading to additional issues including perfusion, functionality, endothelial cell origin and phenotype, and their co-culture with tumor cells. While these goals may seem distant, in fact, they are closer than we realize. Novel techniques involving the separate 3D culture and then combination of nerves (Workman et al., 2017) or neurons (Birey et al., 2017) has led to the connection of cell types and tissue structures previously unattainable. On top of this, automated pipetting, imaging and other robotic strategies will allow for the high-throughput and reproducible output of the model of choice (Kondo et al., 2018).

The final considerations in the engineering of patient-specific microenvironments are to leverage the emerging engineering technologies with relevant characterization technologies. At the cellular level, this includes the identification of intratumoral subclones using next-generation sequencing and combined multi-omics techniques (Chakraborty et al., 2018). Such techniques are key to uncover molecular signatures underlying heterogeneous phenotypes, yet are faced with bioinformatics challenges such as data analysis, interpretation, and multi-technique integration into comprehensive stratifications (Halfter and Mayer, 2017). Such an undertaking will be critical to match tumor subcategories into representatively stratified tumor models for clinical implementation. Next, the characterization of 3D models are often faced with limited high content characterization that prohibit rapid and in-depth analysis in live settings. Again, this will need to be addressed by increasing the capacity of *in situ* localized detection combined with more powerful computational modeling to enable more effective quantification of mechanistic and drug responses in heterogeneous microenvironments (Xu et al., 2014). Ultimately, it is only when combining heterogeneity considerations and working toward the development of comprehensively integrated technologies that we will have a decent chance to reconstitute the complex tumor microenvironment, which is key to understanding individual cancer progression and realistically enable personalized medicine.

## Author Contributions

NB delineated the topic and outline. LB and NB reviewed and evaluated the literature, designed, and wrote the article. DH provided feedback and edited the final article.

### Conflict of Interest Statement

The authors declare that the research was conducted in the absence of any commercial or financial relationships that could be construed as a potential conflict of interest.
